# Mutation-specific peripheral and ER quality control of hERG channel cell-surface expression

**DOI:** 10.1038/s41598-019-42331-6

**Published:** 2019-04-15

**Authors:** Brian Foo, Camille Barbier, Kevin Guo, Jaminie Vasantharuban, Gergely L. Lukacs, Alvin Shrier

**Affiliations:** 1 Department of Physiology, McGill University, Montréal, H3G 1Y6 Québec Canada; 2 Department of Biochemistry, McGill University, Montréal, H3G 1Y6 Québec Canada; 3 Present Address: Institut de Recherches Cliniques de Montréal (IRCM), Montréal, H2W 1R7 Québec Canada; 4 Present Address: Avara Pharmaceutical Services, Boucherville, J4B 7K8 Québec Canada

## Abstract

Impaired functional plasma membrane (PM) expression of the hERG K^+^-channel is associated with Long-QT syndrome type-2 (LQT2) and increased risk of cardiac arrhythmia. Reduced PM-expression is primarily attributed to retention and degradation of misfolded channels by endoplasmic reticulum (ER) protein quality control (QC) systems. However, as the molecular pathogenesis of LQT2 was defined using severely-misfolded hERG variants with limited PM-expression, the potential contribution of post-ER (peripheral) QC pathways to the disease phenotype remains poorly established. Here, we investigate the cellular processing of mildly-misfolded Per-Arnt-Sim (PAS)-domain mutant hERGs, which display incomplete ER-retention and PM-expression defects at physiological temperature. We show that the attenuated PM-expression of hERG is dictated by mutation-specific contributions from both the ER and peripheral QC systems. At the ER, PAS-mutants experience inefficient conformational maturation coupled with rapid ubiquitin-dependent proteasomal degradation. In post-ER compartments, they are rapidly endocytosed from the PM via a ubiquitin-independent mechanism and rapidly targeted for lysosomal degradation. Conformational destabilization underlies aberrant cellular processing at both ER- and post-ER compartments, since conformational correction by a hERG-specific pharmacochaperone or low-temperatures can restore WT-like trafficking. Our results demonstrate that the post-ER QC alone or jointly with the ER QC determines the loss-of-PM-expression phenotype of a subset of LQT2 mutations.

## Introduction

The human ether-a-go-go related gene (hERG) encodes the α-subunit of the Kv11.1 K^+^-channel. In ventricular tissue, hERG is responsible for the rapid delayed rectifier current (I_Kr_), involved in terminal repolarization of the cardiac action potential^1^. Loss of hERG function impairs cardiac repolarization and is associated with Long-QT Syndrome type-2 (LQT2). Prolongation of the cardiac action potential manifests as an extended QT interval on the electrocardiogram and can result in increased risk of torsades-des-pointes arrhythmia and sudden cardiac death^2,3^. LQT2 can arise from loss-of-function mutations in the hERG gene (inherited LQT2) or as an off-target drug effect (acquired LQT2). Interestingly, LQT-associated mutations predominantly act not by impairing channel function, but by impacting folding and conformational stability which in turn leads to recognition and degradation of functional channels by protein quality control (QC) machinery^4–6^.

Cells have evolved numerous QC mechanisms to recognize and dispose of non-native, damaged or aggregated proteins in multiple cellular compartments^7–9^. Newly synthesized membrane proteins are subject to QC at the endoplasmic-reticulum (ER)^10^. Pending the severity of the conformational defect, partially folded proteins can be chaperoned to attain their native conformation before being packaged into COPII transport vesicles for export via the secretory pathway^10^. Irreversibly misfolded polypeptides are disposed via the ER-associated degradation pathway (ERAD) involving the ubiquitin (Ub)-dependent proteasome system (UPS)^10^, or selective autophagy of the isolated ER domain^11,12^.

Premature degradation of misfolded yet partially functional proteins can contribute to the molecular pathology of conformational diseases, including LQT2 and cystic fibrosis^13^. At the ER, folding of nascent hERG involves a network of chaperone interactions, including Hsp70^14^, Hsp90^15^, FKBP38^16^, calnexin^17^, and the Hsp40 family co-chaperones DNAJB12 and DNAJB14^18,19^. hERG channels which fail to adopt a native-like conformation are ubiquitinated (at-least in-part by the CHIP^19^, RFFL^20^ and Trc8^21^ Ub E3 ligases) and retrotranslocated for proteasomal degradation^15,22,23^. This defective ER processing is presumed to underlie the near-complete loss-of-PM-expression phenotype of severely misfolded hERG variants (e.g. G601S, R752W and F805C)^4,5,22^. Alternatively, the WT-hERG conformation may be destabilized by off-target drug-binding in a subset of acquired-LQT2^24–26^. Expression of certain trafficking-defective hERG mutants has also been associated with activation of ER-stress pathways^27,28^ and formation of detergent-insoluble aggregates^27^.

Although it is widely understood that ER QC plays a central role in the proteostasis of integral membrane proteins, there is a growing appreciation that QC mechanisms in post-ER (peripheral) cellular compartments (such as the PM and endosomes in eukaryotic and plant cells) may also play a role^9,29^. We have shown that molecular chaperones can modulate the proteostasis of mutant cystic fibrosis transmembrane conductance regulator (CFTR) at the PM of epithelial cells^30–32^. Partially-redundant E3 ligases (including CHIP^30^, RFFL^32^ and Nedd4/Rsp5) have been implicated in the ubiquitination-dependent degradation of misfolded membrane proteins, including disease-associated variants of CFTR^30^, D4 dopamine receptor, V2 vasopressin receptor^33^ and megalencephalic leukoencephalopathy with subcortical cysts (MLC1)^34^. Poly/multi-mono-ubiquitination acts as an efficient sorting signal for these misfolded PM proteins, triggering rapid internalization and endosomal sorting complex required for transport (ESCRT)-dependent sorting for MVB-lysosomal proteolysis^35,36^.

The PM expression of severely-misfolded hERG mutants (which are normally retained in the ER) can be transiently restored by culturing for extended periods at low-temperature^4^. When returned to physiological temperature, these “rescued” channels are prone to unfolding at the PM, resulting in chaperone-dependent polyubiquitination by the CHIP E3 ligase^37^. Polyubiquitinated channels are rapidly internalized and undergo efficient ESCRT-dependent sorting for lysosomal delivery^6,37^ consistent with the clearance mechanism of other misfolded PM substrates^30,32^. Whether this and/or alternative QC mechanism can recognize hERG variants with limited conformational defects and constitutive ER QC leak remain unknown.

Here, we characterize the cellular processing of mildly-misfolded hERG variants with disease-associated mutations in the cytosolic Per-Arnt-Sim (PAS) domain. Generally, PAS-mutant channels display partial ER-exit and mild/moderate PM-expression defects at physiological temperature. Incomplete ER-retention involved impaired conformational maturation coupled with proteasome-dependent degradation. PAS-mutant channels which constitutively evade the ER QC are rapidly removed from post-ER compartments by a peripheral QC pathway involving rapid endocytosis via a clathrin- and ubiquitin-independent mechanism, impaired endocytic recycling and preferential sorting to lysosomes. Defective processing of PAS-mutant hERG at both the ER and cell-periphery could be rescued by conformational correction with pharmacochaperones or reduced temperature. These results indicate that ER QC and peripheral QC systems both jointly contribute to the mutation-dependent loss-of-expression phenotype in a subset of conformationally defective LQT2 hERG variants.

## Results

### PAS domain mutants as model substrates of hERG quality control

**Figure 1:**
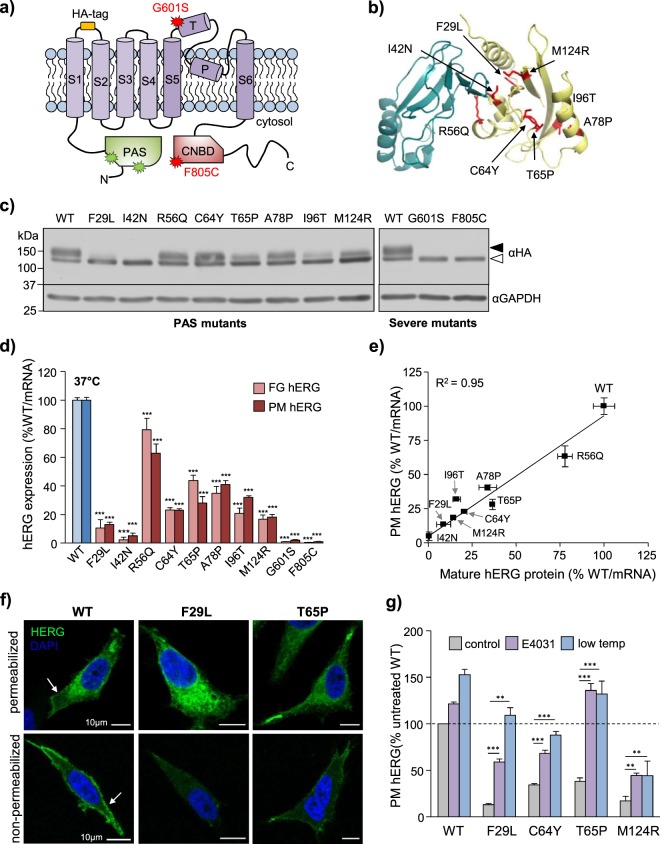
Mutations in the hERG PAS domain produce a range of expression defects. (**a**) Domain structure of hERG1a. Indicated are: Per-Arnt-Sim (PAS) domain, cyclic nucleotide binding domain (CNBD), transmembrane helices (S1-S6), extracellular turret (T), intramembrane pore (P) and engineered extracellular HA-epitope tag. Location of ER-retained mutations (G601S and F805C)^4,37^ and PAS-mutations employed in this study are indicated in red and green, respectively. (**b**) Structural model of the hERG PAS domain (yellow) and CNBD (cyan). PAS domain mutations described here shown in red. hERG cryoEM structure (PDB 5va1) described previously^44^. (**c**) Mutations reduce the expression of complex-glycosylated mature hERG channels. WT, PAS-mutant and severely misfolded (G601S and F805C) hERG stably expressed in HeLa cells and detected by immunoblotting. Immature core-glycosylated (~135 kDa) and mature complex-glycosylated (~155 kDa) hERG indicated with empty and solid arrows, respectively. GAPDH: loading control. Representative immunoblots shown (uncropped images in Supplementary Fig. S12). Solid line: different parts of the same gel. White space: separate gels. (**d**) Quantitative analysis of PAS-mutants expression defect. Mature hERG protein levels and PM-expression were determined by immunoblotting and PM-ELISA, respectively. Expression was normalized to hERG mRNA quantity and expressed as percent of WT. (**e**) Correlation between mature protein and PM hERG expression. Correlation determined by linear regression (R^2^ = 0.95). (**f**) PAS-mutants accumulate in intracellular compartments. PM and cellular hERG immunostained prior to or following fixation and permeabilization. WT-hERG shows strong PM distribution (white arrow) while select PAS mutants (F29L and T65P) are predominantly confined to intracellular compartments. Higher-magnification images and analysis of an additional mutant (M124R) shown in Supplementary Fig. S2. Scale bar: 10 µm, (**g**) Rescue of hERG folding restores PM expression. PM expression of WT and PAS-mutant hERG (F29L, C64Y, T65P and M124R) determined by PM-ELISA following low-temperature incubation (30 °C, 24 h) or treatment with the hERG pharmacochaperone E4031 (10 µM, overnight). Cell-surface expression normalized to mRNA abundance and expressed as percentage relative to untreated WT-hERG. *P < 0.05, **P < 0.01, ***P < 0.001, n.s. = no significant difference (See Methods for explanation of statistical analysis).

The hERG1a protein consists of a 6-helix transmembrane core-region flanked by two cytosolic domains: an N-terminal Per-Arnt-Sim (PAS) domain and a C-terminal cyclic-nucleotide homology binding domain (CNBD)^38^ (Fig. 1a). Disease-associated mutations in the CNBD or transmembrane core (e.g. G601S and F805C) often result in near-complete loss of plasma-membrane (PM)-expression^4,5,39^. Off-target drug binding to the K^+^-conduction pore^25^ (e.g. desipramine)^26^, or loss of cation binding via intracellular K^+^-depletion^24,37^ is also associated with severe conformational destabilization of the WT-channel in a subset of acquired LQT2. Interestingly, while variations within the N-terminal PAS domain can destabilize the isolated region in recombinant expression systems^40,41^, they appear to be generally better tolerated than disruptions to the CNBD or transmembrane core induced by mutations or off-target drug-effect^5^. The PAS domain interacts with the CNBD via an exposed hydrophobic patch to regulate the slow deactivation kinetics of hERG^42^; mutations within or adjacent to this area are linked to accelerated deactivation kinetics in addition to the loss-of-expression phenotype^40–43^. A panel of LQT2-associated mutations lying within (F29L, I42N, R56Q, M124R) or outside (C64Y, T65P, A78P, I96T) the PAS-domain hydrophobic interaction patch were selected for this study (Fig. 1b, Supplementary Table T1)^40,41,44^.

WT and mutant hERG1a containing an HA-epitope tag in the first (S1-S2) extracellular loop^15^ (Fig. 1a) were expressed in HeLa cells by lentivirus-transduction. Nascent hERG1a undergoes N-linked core-glycosylation (CG) in the ER to yield a ~135 kDa polypeptide. Upon folding in the ER^15,45,46^, nascent channels are exported to the Golgi where they undergo additional N-glycan modifications to yield a ~155 kDa complex- or fully-glycosylated (FG) mature channel^6^. The cellular expression of “mature” FG-hERG is assumed to be proportional to that at the cell-surface and has been used as a surrogate indicator of the channel conformational stability^4,5^.

The cellular expression level of mature (FG) hERG variants was compared by quantitative immunoblotting with anti-HA antibody (Ab, Fig. 1c), following normalization for mRNA expression to compensate for variable viral integration efficiency (Supplementary Fig. S1). PAS-mutants display a range of FG-hERG expression defects, ranging from marginal (<30% for R56Q) to profound (>90% for F29L) at physiological temperature (Fig. 1d, Supplementary Fig. S1). Under the same conditions, hERG channels containing mutations in the transmembrane core (G601S) or CNBD (F805C) were undetectable. To confirm that the mature hERG protein abundance detected in immunoblots is reflective of cell-surface expression, hERG PM density was determined using cell-surface ELISA (PM-ELISA). HERG expressing at the surface of intact live cells was detected via the engineered extracellular HA-epitope tag and normalized for mRNA expression (Fig. 1d, Supplementary Fig. S1). PM-ELISA results correlated with those obtained by immunoblot analysis (Fig. 1e, R^2^ = 0.95) and are consistent with the previously-established functional-expression defects determined by whole-cell patch-clamp electrophysiology^5,40,41^.

Subcellular localization of WT-hERG and select PAS-mutants was visualized by immunostaining in conjunction with laser confocal fluorescence microscopy (LCFM). PAS mutants were predominantly confined to intracellular compartments, whereas the WT-hERG was robustly expressed at the PM (Fig. 1f top, Supplementary Fig. S2). Consistent with this observation, selective labelling of cell-surface hERG by indirect immunostaining with anti-HA Ab revealed markedly attenuated PM expression of the PAS mutants (Fig. 1f bottom, Supplementary Fig. S2).

Several hERG-specific pharmacochaperones, including the experimental class III antiarrhythmic E4031, have been identified to enhance the expression of a broad range of hERG variants^4,5^. These compounds bind to a pair of non-conserved aromatic amino acids in the hERG pore (F656 and Y652)^47^ and promote the conformational maturation of nascent channels at the ER^23^. It has been previously reported that PAS-mutations reduce the thermal stability of the isolated PAS-domain^40,41^. To determine whether conformational destabilization underlies the PAS-mutants expression defect, we assessed the impact of pharmacochaperone treatment (10 µM E4031, 18 h) and reduced-temperature (30 °C, 24 h)^4,23^ on hERG PM expression. As previously reported^4,23^, both conditions significantly enhanced PAS-mutant expression as measured by PM-ELISA (Fig. 1g). Taken together, these results demonstrate that mutations in the hERG PAS-domain produce a range of expression defects, which can be at-least partially attributed to conformational-dependent recognition of misfolded channels by protein QC machineries.

### Quality control of PAS-mutant hERGs at the ER

**Figure 2:**
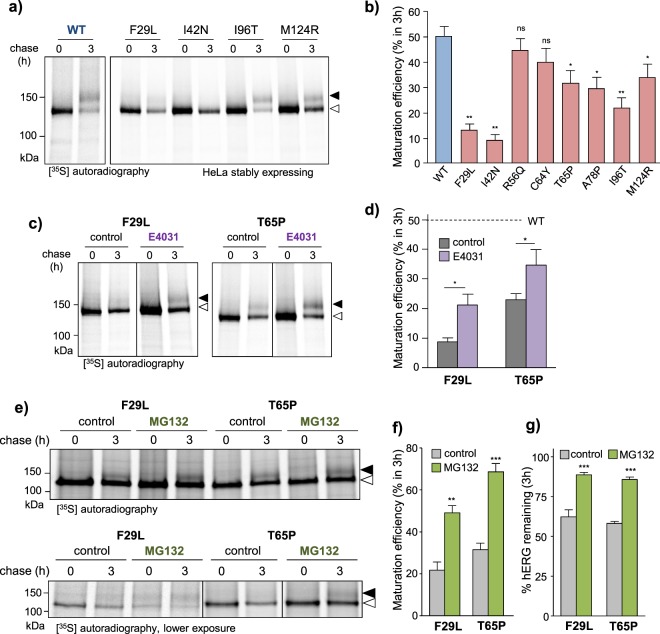
Maturation efficiency and proteasomal degradation of PAS-mutants at the ER. (**a**) Subset of PAS mutants are recognized by the ER QC machinery. Nascent hERG synthesized at the ER were pulse-labeled with [^35^S]-methionine/cysteine and chased for 3 h. hERG was isolated by immunoprecipitation and detected by autoradiography. Mature complex-glycosylated (~155 kDa) and ER-resident core-glycosylated (~135 kDa) hERG indicated by solid and empty arrows, respectively. (**b**) Quantification of WT and select PAS mutant HERG maturation efficiencies. Maturation efficiency calculated as described in Materials and Methods. (**c**,**d**) Pharmacological correction of PAS-mutant folding improves ER processing. Maturation efficiency of T65P and F29L mutant hERG measured by metabolic pulse-chase in the presence of 10 µM E4031. Maturation efficiency calculated as described in Materials and Methods. Mature complex-glycosylated (~155 kDa) and ER-resident core-glycosylated (~135 kDa) hERG indicated by solid and empty arrows, respectively. (**e–g**) PAS-mutants are subject to proteasome-dependent ERAD. Maturation efficiency and metabolic turnover of nascent T65P and F29L mutant hERG measured by metabolic pulse-chase in the presence or absence of proteasomal inhibitor MG132 (10 µM). Maturation efficiency calculated as in (**b**). Turnover expressed as % total (mature + immature) radiolabeled hERG remaining after 3 h chase. *P < 0.05, **P < 0.01, ***P < 0.001, n.s. indicates no significant difference (See Methods for explanation of statistical analysis). Representative images shown (uncropped images in Supplementary Fig. S12). Solid line: different parts of the same autoradiogram. White space: separate autoradiograms.

The near-complete loss-of-PM-expression of severely-misfolded transmembrane/CNBD hERG variants has been attributed to ER-retention and disposal via UPS/ERAD pathways^15,22,23^. The impact of ER QC on PAS-mutant processing and PM expression was evaluated using metabolic pulse-chase technique. Nascent hERG was pulse-labelled with [^35^S]-methionine and [^35^S]-cysteine (30 min, 37 °C), then chased for 3 h in the absence of radioactivity. Conversion of nascent hERG from the immature (~135 kDa) to the mature (~155 kDa) form is presumably dependent on the rate of ER-exit. Consequently, maturation efficiency serves as an indicator of overall ER QC activity encompassing conformational maturation, ER-retention and ERAD. PAS-mutant channels generally displayed decreased maturation efficiency compared to the WT-channel: from 50% ± 4% (refs^15,16^) to ~10–35% over 3 h (Fig. 2a,b).

Overexpression of some ER-retained hERG variants (I593R, G572R and E637K) is associated with an ER-stress response and formation of detergent-insoluble aggregates^27,28^. To assess whether the PAS-mutant partial ER processing defect can be attributed to an ER-stress response, we measured the expression of ER-stress markers including Grp78^48,49^, Grp94^48,49^ and calnexin^50^ by quantitative immunoblotting. No significant increase in the expression of these markers could be observed (Supplementary Fig. S3). Aggregation propensity was evaluated by measuring the partitioning of PAS-mutant hERG into detergent-soluble (1% Triton X-100) and insoluble cell-lysate fractions. Mutant channels were primarily confined to detergent-soluble fractions and did not display increased aggregation propensity (Supplementary Fig. S3).

Having ruled out ER-stress and aggregation, we hypothesized that unfolded/partially-folded PAS-mutant channels are recognized by ER QC in a conformation-dependent manner. To explore this possibility, we evaluated the effect of the pharmacochaperone E4031 (10 µM) on the maturation efficiency of a mild (T65P) and a severe (F29L) mutant. Pharmacological restoration of native-like folding significantly increased the maturation efficiency of F29L- and T65P-hERG from 9 ± 1% to 21 ± 4% and from 23 ± 2% to 35 ± 5% over 3 h, respectively (Fig. 2c,d).

Severely-misfolded hERG variants (e.g. G601S, R752W and F805C) undergo retrotranslocation from the ER-membrane followed by degradation by the UPS^15,22,23^. To determine whether PAS-mutants undergoes similar treatment, we evaluated the impact of proteasomal inhibition on maturation efficiency and metabolic turnover as determined by metabolic pulse-chase. Treatment with proteasomal inhibitor MG132^51^ (10 µM) enhanced the maturation efficiency and delayed metabolic turnover of nascent T65P and F29L hERG (Fig. 2e,f). Taken together, these results suggest that the PAS-mutant PM-expression defect can be, at least in part, attributed to recognition by ER QC pathways involving conformation-dependent ER-retention and proteasomal degradation.

### Peripheral quality control pathways contribute to PAS-mutants expression defect

**Figure 3:**
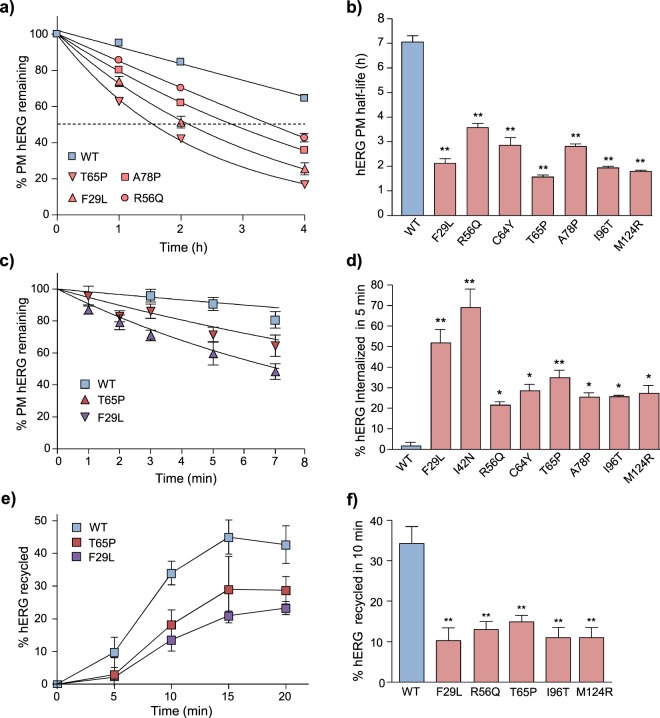
Trafficking of mature PAS-mutants at the PM confers rapid elimination. (**a**) PAS mutant channels are subject to accelerated turnover at the cell-surface. Removal of WT and select PAS-mutant hERG (F29L, R56Q, T65P and A78P) from the cell-surface was measured by PM-ELISA. Turnover kinetics were fit using single-exponential decay functions (solid curves). Similar results obtained for the rest of the PAS mutants (data not shown). (**b**) PM half-life of WT- and mutant hERG calculated from curve-fitting as in (**a**). (**c**) Time course of WT and PAS-mutant (F29L and T65P) internalization. The disappearance of hERG from the cell-surface during a 0–7 minute chase was measured by ELISA and expressed as percent of initial cell-surface hERG remaining. Internalization kinetics fit using single-exponential decay functions. (**d**) Amount of hERG internalized during a 5-minute interval measured by PM-ELISA and expressed as percent of initial cell-surface pool lost. (**e**) Endocytic recycling kinetics of WT, T65P and F29L hERG in HeLa cells. Endosomal hERG pool labelled by Ab capture (20 min at 37 °C); recycling to the PM was measured by sandwich ELISA (0–20 min at 37 °C). Amount of endosomal hERG recycled to the PM expressed as percent of the initial labelled endosomal hERG pool. (**f**) Endocytic recycling of WT and PAS-mutant hERG during a 10-minute chase, measured as in (**e**). *P < 0.05, **P < 0.01, ***P < 0.001, n.s. indicates no significant difference (See Methods for explanation of statistical analysis).

Interestingly, a subset of PAS-mutant channels (e.g. R56Q, C64Y and T65P) mature at rates approaching or equal to that of the WT (~60–100% of the WT-rate, Fig. 2) yet express at only a fraction of that level (~20–60%, Fig. 1). These discrepancies may be due to the action of peripheral QC systems on PAS-mutant channels which evade the ER QC at physiological temperature. To evaluate mutant hERG turnover at the PM, we measured cell-surface stability by PM-ELISA. The PM half-life (T_1/2_) of PAS mutants was reduced from ~7 h to ~1.5–3.5 h (Fig. 3a,b). Our finding parallels the reported rapid metabolic turnover of mature (FG) PAS-mutants upon ER exit block by brefeldin-A^40^.

Clearance of non-native PM proteins has been shown to involve accelerated internalization, impaired endocytic recycling and preferential endosomal sorting complex required for transport (ESCRT)-dependent lysosomal delivery^9,52^; these criteria were examined next. The internalization kinetics of WT-hERG, a moderate (T65P) and a severe (F29L) PAS-mutant was measured by PM-ELISA. The internalization kinetics could be fit with a single-exponential decay function, suggesting that endocytic recycling was negligible during the first 7 min (Fig. 3c). PAS mutants underwent 4–14-fold increased internalization (20–70% per 5 min) relative to the WT (~5% per 5 min, Fig. 3d).

Endocytic recycling of hERG was measured with a sandwich ELISA assay, (described in Materials and Methods) after a 20-minute period labelling of the endocytic hERG pool via anti-HA Ab capture at 37 °C. A severe (F29L) and a mild (T65P) PAS mutant showed impaired endocytic recycling compared to the WT (Fig. 3e). A similar reduction in the recycling of all the PAS mutants could be documented, measured following a 10-minute chase (Fig. 3f).

### Misfolded mature hERG in post-Golgi compartments are delivered to lysosomes

**Figure 4:**
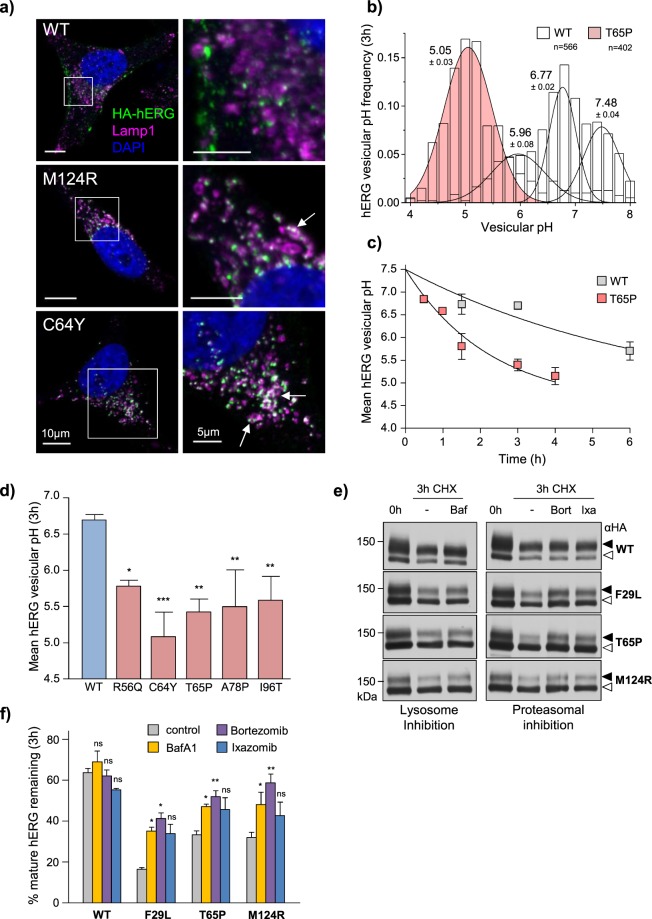
PAS-mutant hERGs are sorted for lysosomal delivery from the cell surface. (**a**) hERG is targeted to LAMP1-positive endo-lysosomal compartments. Endocytic WT, M124R and C64Y hERG pool labelled by Ab capture (15 min at 37 °C) and remaining cell-surface hERG blocked with unconjugated secondary F(ab′)_2_ (1 h on ice). Cells then chased at 37 °C for 3 h prior to fixation. Lysosomal compartments labelled with LAMP1 pAb. hERG (green) and LAMP1 (magenta) staining visualized by LCFM. Whole-cell (scale bar: 10 µm, left) and high-magnification (scale bar: 5 µm, right) images shown. Magnified area indicated by white box. Analysis of additional mutants (F29L, R56Q, T65P) in Supplementary Fig. S4. (**b**) Representative distribution of vesicular pH for WT and T65P hERG containing endocytic vesicles following 3 h chase. Overlay of multi-Gaussian peak-fits shown and mean pH ± SD indicated. N indicates total number of vesicles analyzed in a representative experiment. (**c**) PAS-mutations accelerate hERG endo-lysosomal delivery kinetics. Mean luminal pH of vesicles containing WT or T65P hERG measured by FRIA. Anti-HA Ab and FITC-Fab were bound on ice and FRIA was performed after 1- to 6-h chase. (**d**) Mean luminal pH of vesicles containing WT and PAS-mutant hERG following 3 h chase. (**e**,**f**) Lysosomal activity contributes to degradation of mature hERG proteins. Metabolic stability of WT and PAS-mutants hERG evaluated by immunoblotting following translational inhibition with cycloheximide (CHX, 150 µg/ml). V-ATPase inhibition with Bafilomycin A1 (BafA1, 200 nM), or proteasome inhibition with Bortezomib (Bort, 3 µM) or Ixazomib (Ixa, 3 µM) attenuated the rapid degradation of PAS-mutants. Mature complex-glycosylated (~155 kDa) and ER-resident core-glycosylated (~135 kDa) hERG indicated by solid and empty arrows, respectively. Representative immunoblots shown (uncropped images in Supplementary Fig. S12). Solid line: different parts of the same gel. White space: separate gels. *P < 0.05, **P < 0.01, ***P < 0.001, n.s. = no significant difference (See methods and materials for explanation of statistical analysis).

Lysosomal delivery of PAS-mutant hERG was evaluated by immunofluorescence microscopy. The endocytic hERG pool was labelled by anti-HA Ab capture (15 min, 37 °C) and then chased for 3 h at 37 °C. Colocalization of hERG with the late endosome/lysosomal marker LAMP1 was determined by LCFM. PAS mutant channels showed preferential colocalization with LAMP1 positive vesicles relative to WT hERG (Fig. 4a, Supplementary Fig. S4).

To determine the kinetics of hERG endo-lysosomal delivery, we measured the acidification of hERG containing endocytic vesicles by single cells fluorescence ratiometric image analysis (FRIA)^53^ of vesicular pH (pH_v_). Cell-surface WT and mutant (T65P) hERG were labelled with primary anti-HA and FITC-conjugated secondary F(ab′)_2_ (1 h at room temperature) and then chased for 1–4 h at 37 °C. WT hERG was preferentially retained at early sorting/recycling endosomal compartments (pH_v_ ~6.5–6.8), while the T65P-hERG mutant was rapidly delivered to acidic compartments within 3 h (pH_v_ ~4.5–5.5, Fig. 4b,c). Similar measurements on A78P, R56Q, C64Y, and I96T hERG identified rapid lysosomal delivery as a hallmark of PAS-mutant peripheral processing (Fig. 4d).

To confirm the role of lysosomal proteolysis in the accelerated turnover of PAS-mutants from the periphery, we evaluated the disappearance of complex-glycosylated mature hERG by immunoblotting in the presence of translational inhibitor cycloheximide (CHX, 150 µg/ml). PAS-mutants experienced a greater reduction in mature protein expression following 3 h CHX chase compared to the WT (Fig. 4e,f). Accelerated turnover was attenuated by inhibiting lysosomal acidification and protease activity with Bafilomycin A1^54^ (Baf, Fig. 4e,f) or ammonium chloride^55^ (10 mM, data not shown). Interestingly, proteasomal inhibition with Bortezomib^56^ (3 µM), Ixazomib^57^ (3 µM) or MG132 (10 µM) also attenuated mature hERG turnover as observed previously for some other misfolded and native PM proteins (Fig. 4e,f, data not shown)^58–62^.

### Conformational-selective processing of PAS mutants in post-Golgi compartments

**Figure 5:**
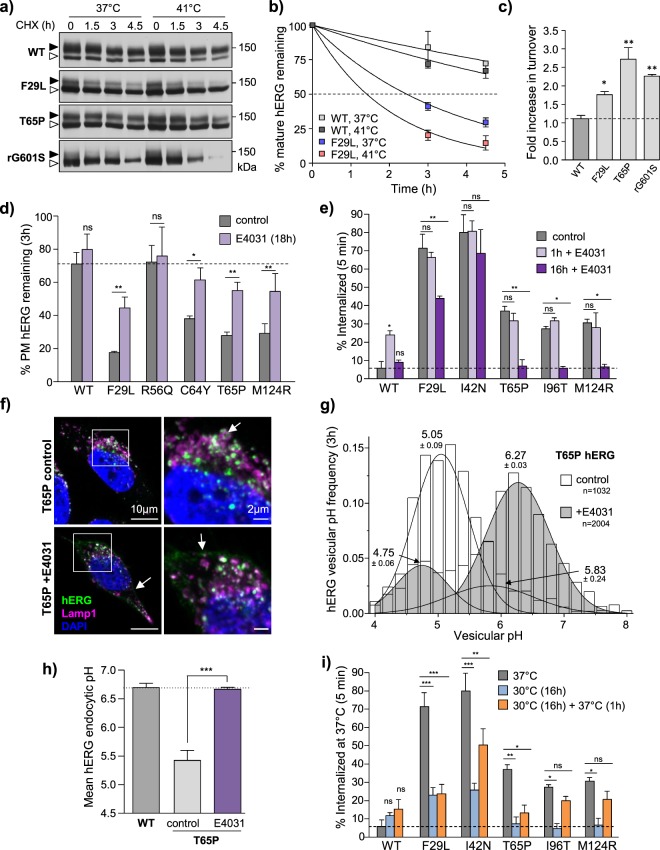
Peripheral quality control engagement is dependent on conformational destabilization. (**a**) Mature hERG is destabilized at elevated temperature. Metabolic stability of mature WT, PAS-mutant (F29L and T65P) or temperature-rescued G601S (48 h at 26 °C, rG601S) hERG evaluated at 37 °C or 41 °C by immunoblotting following translational inhibition with cycloheximide (CHX, 150 µg/ml). Representative immunoblots shown (uncropped images in Supplementary Fig. S12). Solid line: different parts of the same gel. White space: separate gels. (**b**) Turnover kinetics of mature WT and F29L hERG fit using single-exponential decay functions. Similar results obtained for T65P and rG601S hERG (Supplementary Fig. S5). (**c**) Turnover rate-constants determined by curve fitting as in (**b**) and expressed as fold increase relative to 37 °C. (**d**) Pharmacological correction of hERG folding restores cell-surface stability. PM-turnover of WT and select PAS-mutants hERG measured by cell-surface ELISA following overnight (16 h) E4031 treatment (10 µM). (**e**) Pharmacochaperone treatment improves folding of nascent hERG at the ER but does not promote refolding of mature channels at the PM. Internalization of WT and select PAS-mutant hERG measured by PM-ELISA following acute (1 h) or overnight (16 h) E4031 pre-treatment (10 µM). (**f**) Delivery of PM-labelled T65P hERG to LAMP1-positive compartments evaluated by LCFM following 3 h chase. Lysosomal delivery is prevented by overnight pre-treatment with E4031 (10 µM). Whole-cell (scale bar: 10 µm, left) and high-magnification (scale bar: 2 µm, right) images shown. Magnified area indicated by white box. Analysis of WT and additional PAS-mutants in Supplementary Fig. S6. (**g**) Pharmacochaperone pre-treatment prevents endo-lysosomal trafficking of T65P hERG. Representative histogram of T65P hERG vesicular pH following 3 h chase. Overlay of multi-Gaussian peak-fits (mean ± SD) shown. N indicates total number of vesicles evaluated. (**h**) Mean luminal pH of hERG-containing endocytic vesicles measured by FRIA following overnight treatment with E4031 (10 µM) and 3 h chase at 37 °C. (**i**) Subset of temperature-rescued PAS-mutants are resistant to unfolding at physiological temperature. Internalization of WT and PAS-mutant hERG measured by PM-ELISA following low-temperature rescue (30 °C for 24 h) and unfolding (37 °C for 2 h). *P < 0.05, **P < 0.01, ***P < 0.001, n.s. = no significant difference (See Methods for explanation of statistical analysis).

*In-vivo* metabolic turnover of proteins is influenced by multiple factors including conformational stability, secondary structure composition, ligand-binding and protein-protein interactions^63–65^. If the melting temperature (T_m_) of a polypeptide is close 37 °C, structural and metabolic stability may be readily influenced by modulating ambient temperature or ligand/pharmacochaperone binding^66–68^. To assess whether mature complex-glycosylated PAS-mutant channels are conformationally destabilized at/near 37 °C, we compared their metabolic turnovers at elevated temperatures. The turnover of the F29L and T65P hERG was accelerated ~1.5–2.7-fold by increasing ambient temperature to 41 °C, while WT-hERG turnover increased only ~1.1-fold (Fig. 5a–c, Supplementary Fig. S5). The turnover of severely unfolded G601S-hERG^37^ displayed a 2-fold turnover acceleration. Conversely, PAS-mutant hERG PM-stability was significantly increased following conformational correction with E4031 pharmacochaperone treatment (Fig. 5d). Overnight E4031 treatment, but not acute (1 h) exposure attenuated the PAS-mutant rapid internalization phenotype (Fig. 5e). These results suggest that pharmacochaperones act co-translationally to rescue folding of nascent channels consistent with our metabolic pulse-chase results (Fig. 2), but cannot re-fold mature channels following assembly and PM-insertion. Overnight E4031 treatment partially restored WT-like post-endocytic distribution of T65P, F29L and M124R hERG as assayed using immunocolocalization fluorescence microscopy (Fig. 5f, Supplementary Fig. S6). Diversion from acidic endosomal compartments was confirmed for T65P-hERG using FRIA. Overnight E4031 treatment shifted T65P post-endocytic distribution from late endosomes (pH_v_ 5.4 ± 0.1) to early/recycling endosomal compartments (pH 6.67 ± 0.03), mimicking the post-endocytic confinement of WT-hERG (pH_v_ 6.69 ± 0.07) (Fig. 5g,h).

Next, we asked whether “rescued” channel with native-like conformation could be unfolded at the PM. As with pharmacochaperone exposure, incubation at low-temperature for 24 h attenuated the PAS-mutant rapid endocytosis phenotype (Fig. 5i). Returning the cells to 37 °C for 1 h was sufficient to restore rapid endocytosis for some (I42N and T65P), but not all PAS-mutants (Fig. 5i). These mutants likely gained more pronounced thermodynamic and/or kinetic stabilization upon folding at the permissive temperature which renders them resistant to subsequent unfolding.

### PAS mutants are endocytosed by a clathrin- and ubiquitin-independent mechanism

**Figure 6:**
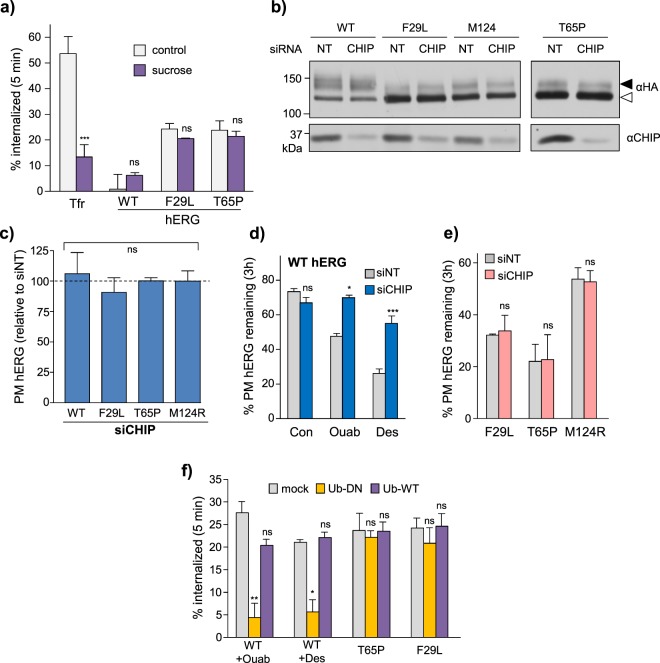
Clathrin- and polyubiquitin-independent peripheral quality control of PAS-mutants. (**a**) PAS-mutants are internalized by a clathrin-independent pathway. Internalization of hERG was measured by cell-surface ELISA in transiently transfected COS-7 cells. Clathrin-dependent internalization was inhibited by incubation in hypertonic media supplemented with 300 mM sucrose (15 min at 37 °C, followed by 45 min at 4 °C). Clathrin-dependent rapid internalization of transferrin receptor (TfR) used as positive control. (**b**) Effect of CHIP ablation on WT and PAS mutants steady-state expression 72 h post-transfection in HeLa cells. CHIP knockdown exceeded 95%. NT: non-target siRNA. Representative immunoblots shown (uncropped images in Supplementary Fig. S12). Solid line: different parts of the same gel. White space: separate gels. Mature complex-glycosylated and core-glycosylated (~135 kDa) hERGs indicated by solid and empty arrows, respectively. (**c**) The CHIP ubiquitin E3 ligase does not influence to the PAS-mutant cell-surface expression. hERG cell-surface density measured by PM-ELISA 48–72 h after transfection with non-target (siNT) or CHIP-specific (siCHIP) siRNA in HeLa cells. (**d**,**e**) CHIP is involved in peripheral quality control of drug-destabilized WT (**d**) but not PAS-mutant hERGs (**e**). Cell-surface turnover of untreated PAS-mutant hERG or WT-hERG unfolded by ouabain-induced intracellular K^+^-depletion (ouab, 300 nM) or by direct desipramine binding (des, 20 µM), monitored by PM-ELISA 48–72 h after transfection with non-target (siNT) or CHIP-specific (siCHIP) siRNA. (**f**) Poly-ubiquitination at the cell-surface is required for internalization of drug-destabilized WT but not PAS-mutant hERG. WT and F29L or T65 hERG were transiently co-expressed with excess wild-type ubiquitin (Ub) or a dominant-negative variant unable to form linked chains (Ub-DN). WT-hERG was unfolded by 2 h pre-treatment with ouabain (Ouab, 300 nM) or desipramine (Des, 10 µM). Overexpression of Ub-DN prevented rapid internalization of the drug-destabilized WT-hERG but had no effect on either PAS mutant. *P < 0.05, **P < 0.01, ***P < 0.001, n.s. = no significant difference (See Methods for explanation of statistical analysis).

Polyubiquitination or multiple-mono ubiquitination by a variety of E3 ligases (e.g. Nedd4/Rsp5, CHIP and RFFL) acts as an internalization and post-endocytic lysosomal sorting signal for PM-proteins in yeast and higher eukaryotes^32,33,69,70^. Ub-dependent internalization of misfolded proteins is initiated by the recruitment of Ub-binding clathrin adaptors (e.g. epsin1 and eps15/eps15R)^9,36,71,72^. To test whether a similar paradigm prevails for the PAS mutants, clathrin-dependent endocytosis was inhibited in hypertonic culture medium supplemented with 300 mM sucrose^36,73^. Hypertonicity inhibited the clathrin-dependent internalization of the transferrin receptor (TfR, Fig. 6a) and a selection of polyubiquitinated CD4-Ub chimeric cargoes^35,36^ (Supplementary Fig. S7). However, the same treatment failed to attenuate the internalization of the T65P- and F29L-hERG mutants (Fig. 6a), suggesting a clathrin-independent endocytic pathway.

Ubiquitination, mediated in-part by the CHIP E3 ligase has been implicated in the PM-removal of the severely misfolded temperature-rescued G601S and F805C hERG mutants^37^, as well as WT-hERG destabilized by intracellular/extracellular K^+^-depletion^37,74^ and direct binding of the antidepressant desipramine^26^. To determine whether CHIP-mediated ubiquitination is also involved in PAS-mutant peripheral QC, we measured the PM density and stability of T65P (mild), M124R (moderate) and F29L (severe) PAS-mutants following siRNA mediated CHIP knockdown. CHIP siRNA has been validated in our HeLa cells^30,37^ and knockdown efficiency was confirmed by immunoblotting (Fig. 6b). CHIP ablation failed to alter either the steady-state cellular expression or stability of PAS-mutant hERG at the PM (Fig. 6c and e). In contrast, CHIP knockdown restored the PM-stability of WT-hERG destabilized by ouabain-induced intracellular K^+^-depletion^37^ or direct binding by desipramine^26^ (Fig. 6d).

CHIP- and clathrin-independent PM quality control of PAS mutant hERG was surprising and may suggest the operation of a ubiquitin-independent QC mechanism. To explore this possibility, we measured the internalization of a mild (F29L) and a severe (T65P) PAS-mutants following overexpression of a mutant ubiquitin substituting arginine for lysine (Ub-AllR). This dominant-negative (DN) variant is incapable of forming poly-Ub chains required for efficient internalization of misfolded PM proteins^35,36^. Intracellular K^+^-depletion or direct binding by the antidepressant desipramine have also been shown to trigger polyubiquitination-dependent degradation of WT-hERG from the PM^26,37^ and were used as positive controls. Overexpression of Ub-AllR but not WT-Ub prevented the internalization of drug-destabilized WT-hERG (Fig. 6f) and a CD4-Ub chimera previously shown to undergo constitutive polyubiquitnation (Supplementary Fig. S7)^35^. Surprisingly, overexpression of Ub-AllR had no effect on the internalization of T65P and F29L hERG (Fig. 6f), suggesting that polyubiquitination is dispensable for their PM-removal.

### PAS mutant hERG ubiquitination is not detectable at post-Golgi compartments

**Figure 7:**
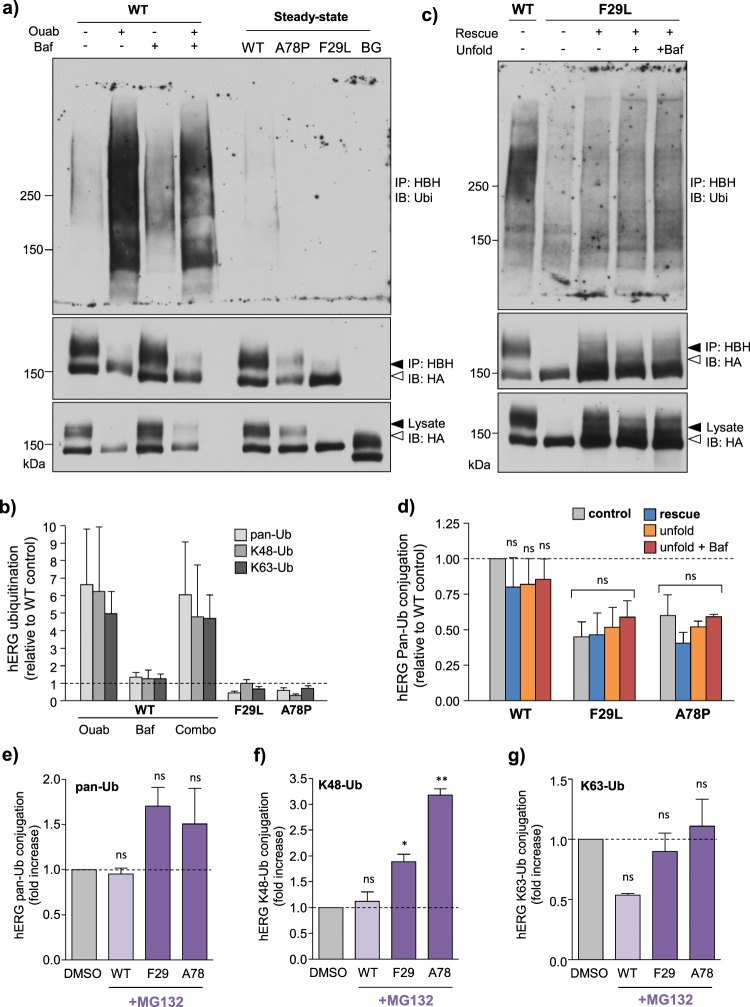
PAS-mutant hERG is negligibly ubiquitinated at post-Golgi compartments. (**a**) PAS mutants are not significantly ubiquitinated under steady-state conditions. hERG-HBH were affinity-isolated on monomeric avidin beads. Ubiquitination detected by immunoblotting with a pan-Ub Ab recognizing mono- and poly-Ub linked chains (P4D1). Destabilization of WT-hERG by acute intracellular K^+^-depletion with ouabain (ouab, 3 h at 300 nM) elicited profound ubiquitination (left). In contrast, PAS-mutant channels did not exhibit a similar increase in ubiquitination (right). Similar results obtained when probed with K48-Ub and K63-Ub chain specific Abs (Supplementary Fig. S10). Non-specific binding assessed in HeLa cells expressing non-HBH-tagged WT-hERG (rightmost lane). Baf: Bafilomycin A1 (200 nM). (**b**) Cells treated as in (**a**) and ubiquitination detected by ELISA. HBH-tagged hERG immobilized onto streptavidin plates and denatured in 8 M urea. Total ubiquitination (mono and polyUb) and K48/K63-Ub linked chains were detected by ELISA, normalized for hERG (HA) signal and expressed as a fraction relative to untreated WT-hERG control. (**c**,**d**) Attenuated PAS-mutant hERG ubiquitination is not dependent on mature protein expression. Cells expressing hERG-HBH were subject to low-temperature rescue (26 °C for 24 h) and subsequent unfolding (37 °C for 3 h) in the presence/absence of Bafilomycin A1 (Baf, 200 nM). hERG was affinity-isolated and total ubiquitination detected by immunoblotting (**c**) or ELISA (**d**) using a pan-Ub Ab (P4D1). Similar results obtained when probed with K48-Ub and K63-Ub chain specific Abs (Supplementary Fig. S10). (**e**–**g**) Proteasomal inhibition causes accumulation of K48-polyubiquitinated PAS-mutant hERG. hERG ubiquitination evaluated by ELISA following acute proteasomal inhibition with MG132 (10 µM for 3 h). Ubiquitination expressed as fold increase over untreated (DMSO) control. Proteasomal inhibition tended to increase total ubiquitination (**e**) and significantly increased K48-polyubiquitination of mutant hERGs (**f**). K63-polyubiquitination was unaffected (**g**). *P < 0.05, **P < 0.01, ***P < 0.001, n.s. = no significant difference (See Methods for explanation of statistical analysis). Full-length anti-Ub immunoblots shown here. Uncropped αHA blots in Supplementary Fig. S12. Solid line: different parts of the same gel. White space: separate gels.

To confirm that PM-retrieval of PAS-mutants is poly- or multimono-ubiquitination independent, we evaluated ubiquitination using biochemical techniques. We engineered a His-Biotin-His (HBH) affinity tag^75^ at the C-terminus of hERG (hERG-HBH, Supplementary Fig. S8). hERG-HBH stably expressed in HeLa cells did not display altered cellular processing or function compared to HA-tagged constructs (Supplementary Fig. S9). HBH-tagged hERG was affinity-purified on monomeric avidin beads under denaturing conditions and channel ubiquitination was evaluated by immunoblotting using a pan-Ub Ab recognizing mono- and poly-Ub adducts, as well as K48-Ub and K63-Ub chain specific Abs (See Methods and Materials). PAS-mutants did not show increased constitutive ubiquitination relative to WT, but rather appeared to be less ubiquitinated (Fig. 7a, Supplementary Fig. S10). In contrast, following unfolding by ouabain-induced intracellular K^+^-depletion, WT-hERG was heavily ubiquitinated consistent with previous results^37^ (Fig. 7a, Supplementary Fig. S10). This ubiquitination pattern was confirmed using an ELISA-based assay. hERG-HBH were bound onto streptavidin-coated multi-well plates under denaturing conditions and ubiquitination was detected with anti-Ub antibodies as described in Methods and Methods (Fig. 7b, Supplementary Fig. S10).

Given the robust ubiquitination of WT-hERG following ouabain treatment despite a comparable reduction in mature hERG abundance, it is unlikely that the limited PAS-mutant ubiquitination of can be explained by decreased expression. To substantiate this conclusion, we evaluated the F29L-hERG ubiquitination following temperature rescue at 30 °C for 24 h (Fig. 1g)^5^. Following rescue, cells were returned to 37 °C and chased in the absence or presence of Bafilomycin A1 (Baf, 200 nM) to impede potential lysosomal degradation of ubiquitinated adducts. This protocol was unable to significantly increase ubiquitination of F29L-hERG, detected by both immunoblotting and ELISA (Fig. 7c,d, Supplementary Fig. S10).

To evaluate whether these PAS-mutants are susceptible to ubiquitination at the ER, HBH-tagged hERG was isolated after proteasome inhibition with MG132 (10 µM) for 3 h, following which ubiquitination was assessed by ELISA. MG132 treatment tended to increase total (poly and mono) ubiquitination of PAS-mutant, but not WT-hERG as detected using a pan-Ub Ab (Fig. 7d). Using chain-specific Abs, we found a significant increase in K48-Ub but not K63-Ub chain modifications on mutant hERG (Fig. 7e,f). K48-Ub chain modification is characteristic of ER QC involving ERAD/UPS^76^, while K63-Ub chains are associated with endocytosis/endosomal sorting^35^. These results suggest that while peripheral QC of PAS-mutants involves a Ub-independent pathway, ER QC likely involves K48-Ub chain dependent ERAD pathways.

### ER and PM QC systems contribution to PAS-mutant hERG expression

**Figure 8:**
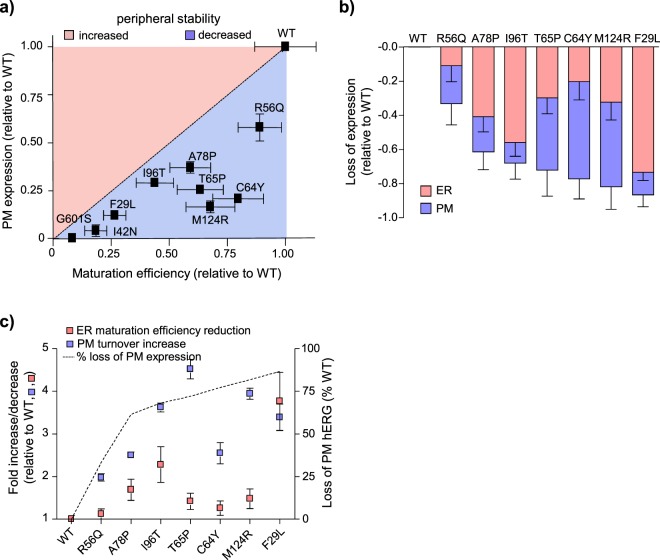
Estimated contributions of ER QC and peripheral QC to hERG loss-of-expression. (**a**) ER processing defect cannot fully account for loss of hERG PM expression. hERG PM expression relative to WT determined by PM-ELISA (Fig. 1) and plotted as a function of relative ER maturation efficiency determined by metabolic pulse-chase (Fig. 2). Discrepancy between the ER processing defect (dotted line) and cell-surface expression (data points) indicates either increased (red) or decreased (blue) PM stability relative to the WT channel. Several mutants (A78P, T65P, M124R, C64Y and R56Q) lie below the dotted line, suggesting the involvement of peripheral QC in defining the PM-expression defect. (**b**) Estimated contributions of ER (red) and peripheral (blue) quality control systems to the total loss of hERG cell-surface expression relative to WT. The predicted loss attributed to each QC pathway is represented as a percentage reduction in PM hERG expression relative to WT. Mutations plotted in order of increasing severity of PM expression defect. (**c**) Differential and mutation-specific sensitivities of ER and peripheral QC machinery. Peripheral QC response (expressed as fold increase in PM turnover) plotted in comparison with the ER QC response (expressed as fold decrease in maturation efficiency). Total expression defect expressed as loss of PM-hERG relative to WT shown on secondary y-axis.

While there is a growing appreciation for the existence of peripheral QC systems acting on misfolded hERG channels at the PM^26,37,40,74^, retention and degradation of nascent channels by ER QC is still generally presumed to be the primary determinant of cell-surface expression^4,5^. Our results, however, suggest that the PM-expression phenotype of certain disease-associated hERG mutants is dictated by the combined action of distinct QC systems acting at the ER and peripheral cellular compartments. If this presumption is correct, the PM expression of these hERG variants should be dictated by the biosynthetic secretion flux at a constant degradation rate from the PM. To test this prediction, we plotted the relative PM expression of PAS-mutant hERGs (determined by PM-ELISA, Fig. 1) against their relative maturation efficiencies (as an estimate of biosynthetic secretion and determined by metabolic pulse-chase, Fig. 2). The PM expression level of several PAS-mutants (A78P, T65P, M124R, C64Y and R56Q) was lower than the predicted value based solely on ER processing efficiency (Fig. 8a). The discrepancy between ER-maturation and PM-expression defects can be used to estimate the peripheral QC contribution to the LQT2-expression phenotype (Fig. 8b, Supplementary Table T2). We find that both ER and peripheral QC contribute to the loss-of-expression phenotype of several PAS-mutants (Fig. 8b). A subset of PAS-mutants (R56Q and C64Y), however, mature at a rate indistinguishable from WT (Fig. 2): the PM-expression defect of these variants appears to be dictated by peripheral proteostasis systems in the absence of ER QC recognition.

The relative contributions of the ER and peripheral QC pathways appear to be highly mutation-specific and independent of the severity of PM expression loss. For example, expression of both R56Q and M124R appear to be predominantly regulated by peripheral QC despite profound differences in PM expression and (presumably) conformational destabilization. Conversely, similar PM expression defect was elicited by the predominantly ER (I96) or PM (T65) associated QC activities. This raises the possibility that the ER and peripheral QC machinery have only partially overlapping substrate recognition criteria and/or thresholds. To explore this, we compared the compartmental-specific cellular processing of each mutant alongside the overall loss of PM expression (Fig. 8c). PAS-mutant peripheral processing exhibited a dynamic response, which generally correlated with mutation severity (~2-fold to ~5-fold increase in turnover relative to WT). However, the ER processing defect was generally both less severe and less dynamic (ranging from WT-like to ~2-fold decrease in maturation efficiency) than the PM QC and, furthermore, did not correlate well with the overall PM expression defect. Only in the case of the most severe PAS mutation (F29L) was there a profound increase in the ER QC response (~4-fold decrease in maturation efficiency). The profound ER processing defect of F29L hERG more closely resembles that seen for other severely compromised PM proteins, including the I42N- and G601S-hERG (Fig. 2b, data not shown) and Δ508-CFTR^77^. These results suggest that ER QC appears to effectively process severely misfolded hERG channels such as F29L and I42N, but it is less effective at recognizing mutants with limited conformational defects. On the other hand, the peripheral QC machinery can recognize all mutations tested in this study and exhibits a dynamic response presumably based on the severity of conformational misfolding.

## Discussion

Recognition and degradation of many misfolded proteins by the ER and peripheral QC systems has been demonstrated, although the degradation signals are not fully understood^9,52^. To the best of our knowledge, this study is one of the first to approximate the relative contributions of the ER and peripheral QC pathways to the loss-of-expression phenotype of a clinically relevant PM protein. We found that ER and peripheral QC systems work in concert to define the PAS-mutant hERG PM-expression: thus establishing the physiological importance of peripheral QC systems in a subset of inherited LQT2 (Fig. 8, Supplementary Table T2). Of particular interest, we describe R56Q and C64Y mutants, which evade ER QC similar to WT (Fig. 2), yet are still recognized as non-native at the periphery (Fig. 3.). This not only underscores the physiological importance of peripheral QC systems but also raises the possibility that the ER and peripheral QC have distinct recognition criteria. It is also possible that the recognition process and/or protein conformation is affected by the local folding environment. A more general implication of this work is that peripheral QC systems could play a significant role in other conformational diseases associated with a similar attenuated (but not abolished) ER processing phenotype.

The involvement of several sequential QC pathways could potentially complicate the therapeutic correction of conformational disease. Peripheral QC pathways could present a significant additional barrier to effective pharmacological correction. For example, the C64Y- and R56Q PAS mutants which display WT-like maturation efficiency and evade the ER QC machinery still exhibit a significant (~30–70%) reduction in PM-expression due to recognition and premature elimination by the peripheral QC (Figs 1d and 2b). On the other hand, we found that a subset of PAS-mutants (F29L, I42N and T65P) were resistant to subsequent unfolding at the PM following low-temperature correction at the ER (Fig. 5i). This result suggests that even transient pharmacological correction of nascent hERG folding and assembly at the ER may result in sustained increase in hERG functional expression.

We were intrigued to observe that proteasomal inhibition prevented turnover of mature, post-Golgi hERG pool (Fig. 4e,f). While the proteasome is well-established to be involved in ER QC via ERAD^10^ (Fig. 2), its quality-control function (if any) at post-ER compartments remains unexplored. Proteasome activity is required for the endocytosis and endo-lysosomal sorting of several PM-proteins including AMPA-type glutamine receptors^58^ and the LDL receptor^59,60^, possibly via degradation of Rab7^61,62^. Alternatively, the proteasome may be involved in cleaving hERG cytosolic domains prior to lysosomal delivery as described for C-terminal truncated CFTR^78^.

Our present study demonstrates that misfolded PAS-mutant hERG are internalized in a clathrin-independent mechanism (Fig. 6a). This is consistent with previous reports that unfolding WT-hERG via extracellular K^+^-depletion or treatment with a cholesterol lowering drug (Probucol) promotes translocation to lipid rafts and subsequent clathrin-independent/caveolin-dependent internalization^79,80^. It is possible that sequestration to lipid rafts and subsequent caveolin-dependent internalization represents a general quality control mechanism for misfolded plasma-membrane proteins; certainly, isolating misfolded cargoes in a dedicated lipid raft compartment would be a useful strategy to prevent disruption of cellular function and cytotoxicity. Ubiquitin-dependent recruitment of membrane proteins to lipid rafts has been described previously^81^ and could be involved in the ubiquitin-dependent degradation of WT-hERG following extracellular K^+^-depletion^82^. Additionally, WT-hERG has been shown to be ubiquitinated by the Nedd4-2 E3 ligase following recruitment to lipid rafts via Caveolin-3^69^. Whether an ubiquitin-independent mechanism exists to recruit misfolded PAS-mutants to lipid rafts and/or trigger caveolin-dependent internalization remains to be seen.

Polyubiquitination, specifically by the CHIP ubiquitin E3 ligase, is involved in the degradation of many misfolded plasma-membrane proteins including disease-associated variants of CFTR (ΔF508), V2 vasopressin receptor, D4 dopaminergic receptor, MLC1, and severely misfolded hERG channels (G601S and F805C mutant and WT-hERG following acute drug treatment or intracellular/extracellular K^+^-depletion)^26,30,33,37,74^. In contrast, the results presented here strongly suggest that the rapid internalization of PAS-mutants from the cell-surface is independent of both CHIP and polyubiquitination. Currently, we cannot rule out the transient ubiquitination of PAS mutants during post-endocytic sorting and lysosomal delivery. Furthermore, while overexpression of dominant negative ubiquitin effectively prevents formation of polyubiquitin linked chains thought necessary for efficient internalization and lysosomal sorting (Supplementary Fig. S7)^35,36^, multiple monoubiquitination remains a possibility. Nonetheless, our inability to detect ubiquitination of F29L hERG even after low-temperature rescue and subsequent thermal unfolding contrasts starkly with our observations for drug-treated WT channels (Fig. 7) and suggests the existence of a distinct recognition and cellular processing mechanism involving dramatically reduced and/or transient ubiquitination, if not complete ubiquitin independence.

It is possible that mutant hERGs are recognized via the exposure of one or more linear sorting motifs. We scanned the hERG protein sequence for a panel of established internalization and lysosomal sorting signals, including tyrosine- and di-leucine-based sorting sequences, as well as caveolin- and ALIX-binding motifs^83–87^. We identified 5 tyrosine-based lysosomal sorting signals located in the hERG cytosolic domains and a KFERQ-related sequence associated with chaperone-mediated autophagy^88,89^, which may be exposed upon misfolding (Supplementary Table T3, Fig. S11). Tyrosine-based sorting motifs promote internalization and sorting to late endosomes/lysosomes by recruiting AP-2 and AP-3 clathrin adaptors, respectively^83,90–92^. However, the endocytosis of misfolded hERG from the cell surface appears to be clathrin-independent (Fig. 6a), making the involvement of tyrosine-based signals and AP-2 unlikely. Aggregation of misfolded channels could also act as a signal for internalization and endosomal sorting^93–96^. We believe that this possibility is unlikely based on the following evidence: (1) PAS-mutants did not display decreased detergent solubility compared to WT-channels (Supplementary Fig. S3) and (2) visualization of immunostained hERG at the cell-surface by confocal microscopy failed to show exacerbated punctate staining which would indicate aggregation (Fig. 1f, Supplementary Fig. S11). However, limited aggregation may not be readily detectable by these methods.

There is a growing appreciation that mutations in different regions of the hERG channel can produce distinct structural defects. Expression analysis and rescue susceptibility of 167 LQT2-linked mutations^5^ showed that N-terminal/PAS mutants were generally amenable to conformational correction (88% of the mutants), consistent with both our and previous observations^40,41^ of a mild expression phenotype. In contrast, C-terminal/CNBD mutants were less amenable to rescue (59%), in line with previous work identifying the CNBD as a key determinant of K^+^-channel structural integrity^39^. Mutations in either cytosolic region were recessive when co-expressed with WT-hERG. In contrast, 39% of pore mutants were resistant to conformational rescue and acted in a dominant-negative manner when co-expressed with the WT-channel. Taken together, these results suggest that mutations in different channel regions may produce distinct structural defects which may influence the recruitment of various QC systems.

Here, we expand on this idea and propose that mutation-specific structural defects may be recognized by distinct proteostasis pathways. This conjecture is based on our observation that PAS-mutant hERG with mild/moderate PM-expression defects were degraded from the periphery via a polyubiquitination-independent mechanism, whereas ER-retained channels destabilized either at the pore (via the G601S mutation or off-target drug treatment) or the CNBD (F805C) are processed in a conventional polyubiquitin-dependent manner (Supplementary Fig. S11)^37^. Whether this differential processing is due to the domain location and/or the severity of the conformational defect remains unknown. Evaluating the peripheral processing phenotype of PAS-mutant channels with a severe loss of PM-expression phenotype (e.g. I31S or Y43C)^40^ and core/CNBD mutations with mild expression defects (e.g. G802R or R784W)^5^ would help us better understand the relationship between conformational disruption, PM-expression and proteostatic processing.

## Materials and Methods

### Plasmids, transfection and expression systems

The WT-hERG-HA expression plasmid has been described previously^37^. PAS-domain mutations (F29L, I42N, R56Q, C64Y, T65P, A78P, I96T and M124R) were generated by site-directed mutagenesis using the Quikchange-XL system (Agilent technologies). All hERG constructs contain an HA-epitope tag in the first (S1-S2) extracellular loop which does not interfere with channel processing or function^15^. The HBH-tandem affinity tag has been described previously^75^. To generate a hERG construct with C-terminal HBH-tag, the stop codon was removed and an AscI restriction site was cloned into the hERG C-terminus by QuikChange mutagenesis. A DNA fragment encoding the HBH sequence (which we have previously described and validated)^32^, GGGS linker and stop codon was generated via overlap-extension PCR and inserted into the hERG vector via the C-terminus AscI and downstream XbaI cut sites (Supplementary Fig. S8). Addition of HBH tag did not alter the expression, cellular processing or function of WT- or PAS-mutant hERG (Supplementary Fig. S9). Expression plasmids encoding N-terminal tagged WT and dominant-negative (Lys-less) ubiquitin constructs and CD4-chimeras have been described previously^35,36^.

HeLa cells constitutively expressing hERG were generated by lentiviral transduction using the pTZV4-CMV-IRES-puro plasmid (Open Biosystems) and maintained in 2 μg/ml puromycin selection. Lentivirus was produced using the Lenti-X system (Takara Bio, USA) as described previously^33,37^. Transient transfection of COS-7 cells was performed using GeneJuice transfection reagent (EMD Milipore) according to manufacturer’s protocols. N-terminal tagged WT and dominant-negative (Lys-less) ubiquitin variants and hERG/CD4 cargoes were cotransfected at a 4:1 cDNA mass ratio and assayed 48–72 h post-transfection as described previously^35,36^.

Small-interfering RNA (siRNA) were purchased from Qiagen. siRNA were transfected into HeLa cells at a final concentration of 50 nM using RNAiMAX transfection reagent (ThermoFisher) according to manufacturer protocol and assayed 72 h post-transfection. Single-sequence siRNA against CHIP (SI00081977, accenssion NM_005861) and non-target siRNA (SI03650318) were validated previously^30,37^.

### Detection of hERG mRNA transcript levels

Cellular mRNA was extracted and purified from HeLa cells using RNeasy RNA isolation kits (Qiagen). Equal quantities of mRNA were reverse-transcribed into cDNA using QuantiTech RT kit (Qiagen) and amplified using SYBR advantage qPCR premix (Clonetech). hERG mRNA content normalized for that of GAPDH. Non-template control and untransfected (parental) cells used for GAPDH and hERG background, respectively. hERG and GAPDH-specific primers were designed using the NCBI primer design tool and listed below.

hERG forward: GGCCAGAGCCGTAAGTTCAT

hERG reverse: TGCAGGAAGTCGCAGGTG

GAPDH forward: CATGAGAAGTATGACAACAGCCT

GAPDH reverse: AGTCCTTCCACGATACCAAAGT

### Western blotting and protein analysis

For immunoblotting, hERG expressing Hela cells were solubilized in Triton X-100 lysis buffer (1% Triton X-100, 25 mM Tris-Cl, 150 mM NaCl, 10 µM leupeptin,10 µM pepstatin, 1 mM PMSF, pH 7.4) for 10 minutes on ice. Detergent-insoluble cellular debris was cleared by centrifugation at 18,000 g (15 minutes, 4 °C). The following antibodies and concentration were used for Western blotting: monoclonal anti-HA (1:1000, clone MMS101R, Covance), polyclonal anti-calnexin (1:2000, Abcam), polyclonal anti-Grp78 (1:4000, Stressmarq), monoclonal anti-GAPDH (1:25,000, clone 6C5, Abcam), monoclonal anti-Grp94 (1:2000, clone 9G10, Santa-Cruz), monoclonal anti-ubiquitin (1:100, clone P4D1, Abcam), monoclonal K48-chain ubiquitin (1:200, clone APU2, Abcam) and monoclonal K63-chain ubiquitin (1:200, clone APU3, Abcam). HRP-conjugated secondary IgG (sheep anti-mouse, donkey anti-rabbit and goat anti-rat, GE Healthcare) were used at 1:1000–1:2000 dilution and detected using SuperSignal ECL chemiluminescent substrate (Thermofisher) on autoradiography film. Band intensities were quantified using ImageJ image analysis software (NIH, USA). Steady-state expression of mature hERG was normalized to mRNA levels and expressed as a percent relative to WT. hERG metabolic stability was determined by immunoblotting following translational inhibition with cycloheximide (150 µg/ml, Sigma) and expressed as % remaining. Representative immunoblots shown. Full-length (uncropped) immunoblots in Supplementary Figs S12 and S13. Solid line: different parts of the same gel. White space: separate gels. All antibodies used for immunoblotting have been previously described^37,97^.

### Determination of hERG maturation efficiency by metabolic pulse-chase

The maturation efficiency of hERG at the ER was determined using metabolic pulse-chase technique. Prior to radio-labelling, cells were cultured in methionine/cysteine free media for 45 minutes. MG132 and E4031 were included during met/cys depletion and maintained throughout the chase. Newly synthesized hERG was labelled with EasyTag [^35^S] Met/Cys labelling mixture (30 minutes, 1 µCi/ml; PerkinElmer) and chased for 0 or 3 hours in media supplemented with 2 mM unlabelled Met/Cys. Cells were solubilized in Triton X-100 lysis buffer with protease inhibitors as described above. hERG was isolated by immune-precipitation with previously-validated^21,37^ polyclonal anti-hERG Ab against a C-terminus epitope (1:200; Alomone labs, Israel) on Protein-G beads (Life Technologies). Core-glycosylated and fully-glycosylated hERG were separated using SDS gel electrophoresis. Autoradiography was performed using BAS storage phosphor screens (Fujifilm Japan) or X-ray autoradiography film. Band intensities were quantified using ImageQuant image analysis software (GE healthcare). Maturation efficiency was calculated as FG_3_/(CG_0_ − CG_3_) and expressed as a percentage, where FG_3_ represents the amount of mature complex-glycosylated hERG at 3 h, and CG_0_ and CG_3_ represents the immature core-glycosylated hERG pool prior to or following 3 h chase, respectively. Representative autoradiograms shown. Full-length (uncropped) autoradiograms in Supplementary Fig. S12. Solid line: different parts of the same autoradiogram. White space: separate autoradiograms.

### Measurement of hERG cell-surface density, internalization and metabolic stability

hERG was detected at the PM using the extracellular HA-epitope tag in conjunction with live cell-surface ELISA techniques as described previously^37^. Briefly, the extracellular HA-tag was labelled with mouse monoclonal anti-HA antibody and detected with HRP-conjugated secondary F(ab′)_2_ (Molecular Probes, Eugene OR). HRP detection was done using either Amplex Red fluorogenic substrate or SuperSignal ECL chemiluminescent substrate (Thermofisher). Fluorescence and chemiluminescent signals were measured in quadruplicate samples using Tecan Infinite M1000 (Tecan Group, Switzerland) or Wallac Victor^3^ (PerkinElmer) plate readers, respectively. A non-specific isotype control primary antibody was used to determine the background signal. Steady-state cell-surface hERG levels were normalized to hERG mRNA levels and expressed as a percent relative to WT.

To determine the rate of internalization and metabolic turnover from the cell-surface, cells were chased at 37 °C for 1–10 minutes or 1.5–6 hours, respectively, following primary Ab binding. The kinetics of hERG internalization and metabolic turnover from the cell-surface were fit using single-phase exponential decay functions. All antibodies used for PM-ELISA have been previously described^30,33,37^.

### Endocytic recycling

hERG endocytic recycling was measured using a modified sandwich cell-surface ELISA assay described previously^37^. Briefly, cell-surface hERG was labelled with anti-HA primary antibody (1 h, on ice). Ab-hERG complexes were then internalized for 20 minutes at 37 °C; complexes remaining on the cell surface were then blocked with mouse monovalent F(ab′)_2_ fragments (1:100; Jackson ImmunoResearch Laboratories, 1 h on ice). Recycling of the internalized Ab-hERG complexes was enabled by incubating cells at 37 °C for 0–20 minutes. Exocytosed Ab-hERG complexes were detected with HRP-conjugated secondary F’(ab)_2_ as described above. Background signal was measured using a non-specific primary Ab as described above. Blocking efficiency with mouse monovalent F(ab′)_2_ fragment was determined to be over 95% (data not shown). The size of the endocytic hERG pool following 20-minute incubation at 37 °C was measured in parallel, with recycling efficiency being expressed as percent of this pool.

### Measurement of hERG vesicular pH by fluorescence ratio imaging analysis (FRIA)

The measurement of endocytic vesicular pH by fluorescence ratiometric imaging has been described in detail by Barriere and Lukacs^53^. Cell-surface hERG were sequentially labelled on ice with mouse anti-HA primary (1:1000) and FITC-conjugated goat anti-mouse F(ab′)_2_ secondary (1:1000). Internalization and subsequent endocytic trafficking was enabled by chasing at 37 °C for the indicated time. Cellular trafficking was halted by cooling cells to 4 °C and FRIA was performed on a Zeiss Observer Z1 inverted fluorescence microscope (Carl Zeiss MicroImaging) equipped with a X-Cite 120Q fluorescence illumination system (Lumen Dynamics Group, Canada) and Evolve 512 EM CCD camera (Photometrics Technology). The acquisition was carried out at room-temperature, with excitation at 495 ± 5 nm and 440 ± 10 nm and detection using a 535 ± 25 nm emission filter. Data was analyzed using MetaFluor image analysis software (Molecular Devices, Canada). Individual vesicular pH measurements were collected from multiple images to generate a histogram of vesicular pH distribution. Histograms were fit with multiple Gaussian distribution using Origin graphing and analysis software (OriginLab) and mean endocytic pH values were calculated as the weighted average of the peaks. All antibodies used for FRIA have been previously described^37^.

### Immunostaining

HeLa cells expressing hERG were cultured on glass cover slips. Cell-surface hERG was labelled with monoclonal anti-HA Ab (1:1000, on ice) prior to fixation with 4% paraformaldehyde (15 minutes at room temperature). Cell-surface Ab-hERG complexes were labelled with Alexa-488 conjugated goat anti-mouse F(ab′)_2_ at room temperature (1:1000; Molecular Probes). hERG post-endocytic fate was visualized using an anti-HA Ab capture assay. Early endocytic hERG pools were labelled by continuous anti-HA Ab capture for 15 minutes at 37 °C. The cell surface hERG pool was then blocked with mouse monovalent F(ab′)_2_ fragment on ice (1:100; Jackson). The internalized hERG pool was chased for the indicated time at 37 °C prior to fixation with 4% paraformaldehyde. Cells were permeabilized with 0.05% saponin and intracellular Ab-hERG complexes fluorescently labelled with Alexa-488 conjugated goat anti-mouse F(ab′)_2_ at room temperature (1:1000; Molecular Probes). Lysosomes were labelled with polyclonal rabbit anti-LAMP1 Ab (1:1000; Abcam) and Alexa-555 conjugated goat anti-rabbit F(ab′)_2_ (1:1000; Molecular Probes). Confocal images were taken on a LSM780 microscope (Carl Zeiss MicroImaging) equipped with a Plan Apochromat 63x/NA 1.4 objective in multitrack mode. Representative single optical sections are shown. Antibodies used for immunostaining have been previously described^37^.

### Measurement of hERG ubiquitination

Ubiquitination of WT- and mutant HBH-tagged hERG variants was detected by either immunoblotting or ELISA on affinity-purified channels. For immunoblot analysis, cells were lysed in 1% Triton lysis buffer as described above, with additional protease and deubiquitinating enzyme (DUb) inhibitors (20 µM MG132, 5 mM NEM, 10 µM PR-619). Lysates were incubated with 20 µl/ml BcMag Monomeric Avidin Magnetic beads for 1 h at 4 °C (Bioclone, San Diego USA). Following binding, beads were washed with washing buffer (0.1% NP40 in PBS) followed by denaturation (8 M urea, 0.1% NP40 in PBS) for 5 minutes at room temperature to disrupt hERG-protein complexes. Bound hERG then eluted by incubation with 5x Laemmli sample buffer supplemented with 6 mM free biotin. hERG, total mono/poly-ubiquitin (Pan-Ub) and K48/K63 poly-Ub linked chains detected by immunoblotting using antibodies described above.

To detect hERG ubiquitination by ELISA, cells were lysed in 1% Triton lysis buffer in the presence of additional protease and DUb inhibitors as described above. Streptavidin-coated 96-well plates (ThermoFisher) were blocked (0.5% BSA, 0.1% NP40 in PBS, 30 minutes on ice) prior to immobilization of hERG-HBH (90 minutes on ice). Following binding, plates were washed (0.1% NP40 in PBS) and bound proteins were denatured with urea (8 M urea, 0.1% NP40 in PBS). Immobilized hERG, total mono/poly-ubiquitin (Pan-Ub) and K48/K63 poly-Ub linked chains labelled with primary antibody and HRP-conjugated secondary F(ab′)_2_ antibody fragment using antibodies described above. HRP signal detected using SuperSignal ECL chemiluminescent substrate (Thermofisher) and measured using Wallac Victor^3^ (PerkinElmer) plate reader. A non-specific isotype control primary antibody was used to determine the background signal.

### Statistical analysis

All data were analyzed using Graphpad Prism (Graphpad Software) unless otherwise stated. For multiple comparisons against a single control (e.g. multiple mutants vs. WT), statistical significance was determined using one-way ANOVA with Dunnett’s post-hoc test against control. For multiple comparisons against several controls (e.g. multiple mutants following drug treatment vs. untreated controls), statistical significance was determined using one-way ANOVA with Bonferroni correction for multiple comparison. For multiple comparisons against a hypothetical control value (e.g. data expressed as a fraction of relative to WT), significance was determined using one-sample T-test with Bonferroni correction for multiple comparisons. Data represented as mean ± SEM from at least 3 independent experiments unless otherwise indicated. Significance indicated as *P < 0.05, **P < 0.01, ***P < 0.001 and n.s. indicates no significant difference. Values tested for significant differences indicted by brackets only where not immediately obvious. Direct correlations were evaluated using linear regression. Half-lives and rate-constants of decay were determined by fitting a single-exponential decay function assuming a plateau of 0 (i.e. eventual complete degradation). Multiple-Gaussian fits for hERG vesicular pH performed using Origin (OriginLab).

### Presentation of immunoblots, autoradiograms, microscopy images and structural model

Cropped sections of representative immunoblots are shown in the main text. Uncropped immunoblots for main-text and supplemental figures available in Supplementary Figs S12 and S13, respectively. Solid lines indicate different parts of the same gel; white space indicates separate gels. Dotted line indicated where membrane was cut to facilitate probing for different proteins. Where appropriate, mature (complex-glycosylated) and immature (core-glycosylated) hERG indicated by solid and empty arrow, respectively. Asterisk (*) indicated non-specific band. Densitometric quantification of immunoblots was done using at-least 2 exposures from the same gel. Representative confocal images from a sample of at-least 15 images are shown.

The hERG cryo-EM structure has been previously published^44^. Structural coordinates obtained from the Protein Data Bank (5va1) and visualized using PyMol (Schrödinger, NY, USA). Polypeptide motifs in the hERG protein sequence were identified using the ScanProSite online tool (Swiss Institute of Bioinformatics via expasy.org).

## Supplementary information

Supplementary Information

## Data Availability

Any materials, data and associated protocols described in this work are available upon request without undue qualifications.
