# Supplementary material for: Mutation-specific peripheral and ER quality control of hERG channel cell-surface expression
Source: Sci Rep. 2019 Apr 15;9:6066. doi: 10.1038/s41598-019-42331-6 (PMC6465299; doi:10.1038/s41598-019-42331-6)
Supplement: Supplementary file 1 — Supplementary Information [file 41598_2019_42331_MOESM1_ESM.pdf]

Mutation-specific peripheral and ER quality control of hERG channel cell-surface expression

Supplementary Information

Brian Foo[1], Camille Barbier[1], Kevin Guo[1,2,#], Jaminie Vasantharuban[1,&],
Gergely L. Lukacs[1,2,†] and *Alvin Shrier[1,†]

[1]Department of Physiology and [2]Biochemistry McGill University,
Montréal, Québec, Canada, H3G 1Y6

***Corresponding author**:
Dr. A. Shrier: Department of Physiology, Bellini Building Room 165, McGill University, 3649 Promenade Sir William Osler, Montréal, Québec, Canada H3G 0B1.
Email: alvin.shrier@mcgill.ca

[†]Contributed equally to this work

[#]Current corresponding address: Institut de Recherches Cliniques de Montréal (IRCM), 110 Avenue des Pins, Montréal, Québec, Canada H2W 1R7.

[&]Current corresponding address: Avara Pharmaceutical Services, 145 rue Jules léger, Boucherville, Québec, Canada J4B 7K8.

Supplementary methods

Detection of detergent-insoluble hERG fraction

hERG expressing Hela cells were solubilized in Triton X-100 lysis buffer (1% Triton X-100, 25mM Tris-Cl, 150mM NaCl, 10μM leupeptin,10μM pepstatin and 1mM PMSF, pH 7.4) for 10 minutes on ice. Cell lysate was centrifuged at 18,000g (15 minutes, 4°C) or 100,000g (30 minutes, 4°C) to isolate the detergent-insoluble cellular fraction. Pellets were resuspended in 1% Triton (1% Triton X-100, 1mM PMSF, 1mM $MgCl_2$, 50mM TRIS-HCl pH 8.0) and chromosomal DNA was degraded using benzonase (Sigma, 50 units/ml, 30 minutes at 37°C). Detergent-insoluble proteins were then resolubilized by adding 3 volumes of 8M urea buffer (8M urea, 30mM EDTA, 70mM DTT) for a final urea concentration of 6M. Both detergent-soluble and detergent-insoluble fractions were denatured in 2X Laemmli sample buffer (4% SDS, 192.5 mg/ml DDT, 3.5% glycerol, 0.004% bromphenol blue, 0.125 M Tris HCl pH 6.8) prior to SDS-PAGE.

Measurement of CD4 internalization

CD4 internalization was detected at the PM by cell-surface ELISA as described previously[1,2]. CD4 constructs were transiently transfected into COS-7 cells 3 days before the experiment. In experiments involving co-expression of ubiquitin variants, CD4 and Ub plasmids were co-transfected at a 1:4 mass ratio. Cell-surface CD4 was labelled with a previously-validated[1,2] mouse monoclonal antibody recognizing an extracellular epitope (clone OKT4, Thermofisher) and chased at 37°C for 5 minutes. Remaining cell-surface CD4 was labelled with HRP-conjugated secondary anti-mouse $F(ab')_2$ antibody fragment (Molecular Probes, Eugene OR) and detected using Amplex Red fluorogenic substrate (Thermofisher). Non-transfected cells were used to determine the background signal.

Patch-clamp electrophysiology

Cells were plated on 8mm coverslips or 35mm plastic tissue culture dishes (pre-coated with 0.1 g/mL poly-l-lysine) and incubated for 3h at 37°C to allow for sufficient cellular adhesion. Next, cells were placed in the perfusion chamber of an inverted microscope (Zeiss Axiovert S100TV or 135) and perfused at a rate of 1-2 mL/min with an extracellular solution containing 135mM NaCl, 5mM KCl, 2mM $CaCl_2$, 1mM $MgCl_2$, and 10mM HEPES (pH 7.4 with NaOH, ~285mOsm). Patch pipettes were fabricated using borosilicate glass capillaries (Warner Instruments, Hamden, CT) and a microprocessor-controlled, multi-stage micropipette puller (P97, Sutter Instruments), and subsequently fire polished (CPM-2, ALA Scientific instruments, Farmingdale, NY). Pipettes with

resistances of 1.5 - 3MΩ were backfilled with a pipette solution containing 135mM KCl, 5mM EGTA, 1mM $MgCl_2$, and 10mM HEPES (pH 7.2 with KOH, ~285mOsm). The liquid junction potential (LJP) between the extracellular solution and pipette solution (1.5mV) was corrected offline using the formula $V_{membrane} = V_{Pipette} - V_{LJP}$ as described previously[3]. All experiments were performed at room temperature (~21°C), and all cells were perfused with extracellular solution for 10 minutes prior to experimentation to ensure complete replacement of cellular media.

Whole-cell currents were recorded using an Axopatch 200B amplifier (Axon Instruments, Sunnyvale, CA) coupled to a CV 203BU headstage (Axon Instruments) or a VE-2 amplifier (Alembic Instruments, Montreal, QC) coupled to a VE-2 headstage (Alembic Instruments). Command pulses were generated by a Digidata 1440A (Axon Instruments) via pClamp 10.4 software or by a Digidata 1322A digitizer (Axon Instruments) via pClamp 10.2 software. Data were acquired at 20kHz and low pass filtered at 2kHz or 3kHz.

Upon the formation of a GΩ seal and prior to membrane rupture, currents were corrected for pipette (fast) capacitance. Once ruptured, cell capacitance (picofarad; pF) was determined using a 30ms, 10mV depolarizing pulse from a holding potential of -80mV, at 2Hz. Currents were corrected for whole-cell capacitance and series resistance compensated to 80% (Axopatch) or ~100% (Alembic). All presented cells have access resistances below 15MΩ, membrane capacitances greater than 10pF, and reversal potentials between -70mV and -90mV (determined offline). Cells that did not express hERG, or were characterized as “low expressers” (i.e. 5% of or less of mean current), were excluded. Representative traces are presented in pA, current-voltage (I-V) relationships in pA/pF, and time-constants (τ) in ms.

Voltage protocols and analysis

The steady-state I-V relationship and steady-state activation curve were determined using a two-step protocol. Cells were held at -80mV, stepped in +10mV depolarizing pulses from -60mV to +50mV for 4 seconds (P1 pulse), and subsequently stepped to -50mV for 4.5 seconds (P2 pulse). The full series of currents obtained using this protocol for HA-hERG-HbH and HA-hERG control are shown in Supplementary Fig. S9. The steady-state I-V relationship was obtained by plotting the peak current at the end of the P1 pulse against the P1 pulse voltage. The steady-state activation curve was obtained by plotting the peak tail currents generated at the onset of the P2 pulse against the previous P1 pulse voltage. These values were normalized values and fit with the Boltzmann sigmoidal equation.

Supplementary Tables

Table T1: Characteristics of selected PAS-domain mutations
Properties of LQT-associated PAS-domain mutations used in this study. Mutations categorized based on their location either at the PAS-CNBD interface or elsewhere in the PAS domain (Fig. 1). Deactivation kinetic data previously described[4] and validated for a subset of mutants (R56Q, C64Y and M124R, data not shown).

Mutation	Location	Deactivation
F29L	Both	Fast
I42N	Interface	Fast
R56Q	Interface	Fast
C64Y	Internal	WT-like
T65P	Internal	Mild acceleration
A78P	Internal	WT-like
I96T	Internal	WT-like
M124R	Interface	Fast

Table T2: hERG cellular processing defects and their contribution to PM-expression
Summary of empirically determined hERG PM-expression and processing defects (left) and estimated contribution of ER and peripheral QC systems to the disease phenotype (right). PM-expression determined by ELISA (Fig. 1) and expressed as fraction relative to WT following normalization for mRNA content. Maturation efficiency determined by metabolic pulse-chase (Fig. 2) and expressed as a fraction relative to WT. It is assumed that maturation during the 3h chase is proportional to biosynthetic flux. PM-turnover measured by PM-ELISA; rate-constants of degradation determined by curve-fitting as in Fig. 3 and are expressed as fold-increase relative to WT. The ER QC contribution to overall loss-of-expression is assumed to be proportional to the ER-maturation efficiency defect; the estimated peripheral QC contribution was calculated as the difference between the ER-maturation defect and total expression defect as in Fig. 8. The estimated contributions of peripheral and ER QC to hERG loss-of-expression are represented as % of the WT. Data represented as mean ± SEM from at-least 3 independent experiments.

	**Cellular processing phenotype** (relative to WT)			**Compartmental contribution** (% reduction relative to WT)	
Mutant	PM expression	Maturation efficiency	PM turnover rate	Peripheral QC	ER QC
WT	1	1	1	0	0
R56Q	0.67 ±0.03	0.89 ±0.09	1.98 ±0.09	-22 ±12	-11 ±9
A78P	0.38 ±0.02	0.59 ±0.09	2.50 ±0.08	-21 ±10	-41 ±8
I96T	0.31 ±0.01	0.43 ±0.08	3.6 ±0.1	-12 ±9	-56 ±8
T65P	0.28 ±0.06	0.70 ±0.09	4.5 ±0.2	-42 ±15	-30 ±9
C64Y	0.23 ±0.01	0.79 ±0.1	2.5 ±0.2	-57 ±12	-20 ±11
M124R	0.18 ±0.03	0.68 ±0.1	3.9 ±0.1	-50 ±13	-32 ±10
F29L	0.13 ±0.02	0.26 ±0.05	3.4 ±0.3	-13 ±7	-73 ±5

Table T3: hERG contains several sorting sequences in cytosolic regions
Tyrosine-based sorting signals and KFERQ-related motif present in the hERG cytosolic domains. The peptide motif is underlined and shown in the context of the five-adjacent flanking amino acids. Polypeptide motifs in the hERG protein sequence were identified using the ScanProSite online tool (Swiss Institute of Bioinformatics via expasy.org).

	**Sequence**	**Motif**	**Location**
1)	C E L C G Y S R A E V M Q R	Tyrosine-based	54-57 (PAS)
2)	S D L V R Y R T I S K I P Q	Tyrosine-based	327-330 (PAS)
3)	E P L N L Y A R P G K S N G	Tyrosine-based	812-815 (CNBD)
4)	V R A L T Y C D L H K I H R	Tyrosine-based	827-830 (CNBD)
5)	E V L D M Y P E F S D H F W	Tyrosine-based	845-848 (CNBD)
6)	D T I I R K F E G Q S R K F I	KFERQ-related	21-25 (PAS)

Supplementary References

1 Barriere, H., Nemes, C., Du, K. & Lukacs, G. L. Plasticity of polyubiquitin recognition as lysosomal targeting signals by the endosomal sorting machinery. *Mol Biol Cell* **18**, 3952-3965, doi:10.1091/mbc.E07-07-0678 (2007).

2 Barriere, H. *et al.* Molecular basis of oligoubiquitin-dependent internalization of membrane proteins in Mammalian cells. *Traffic* **7**, 282-297, doi:10.1111/j.1600-0854.2006.00384.x (2006).

3 Neher, E. Correction for liquid junction potentials in patch clamp experiments. *Methods Enzymol* **207**, 123-131 (1992).

4 Ke, Y. *et al.* Trafficking defects in PAS domain mutant Kv11.1 channels: roles of reduced domain stability and altered domain-domain interactions. *Biochem J* **454**, 69-77, doi:10.1042/BJ20130328 (2013).

5 Ficker, E., Dennis, A. T., Wang, L. & Brown, A. M. Role of the cytosolic chaperones Hsp70 and Hsp90 in maturation of the cardiac potassium channel HERG. *Circ Res* **92**, e87-100, doi:10.1161/01.RES.0000079028.31393.15 (2003).

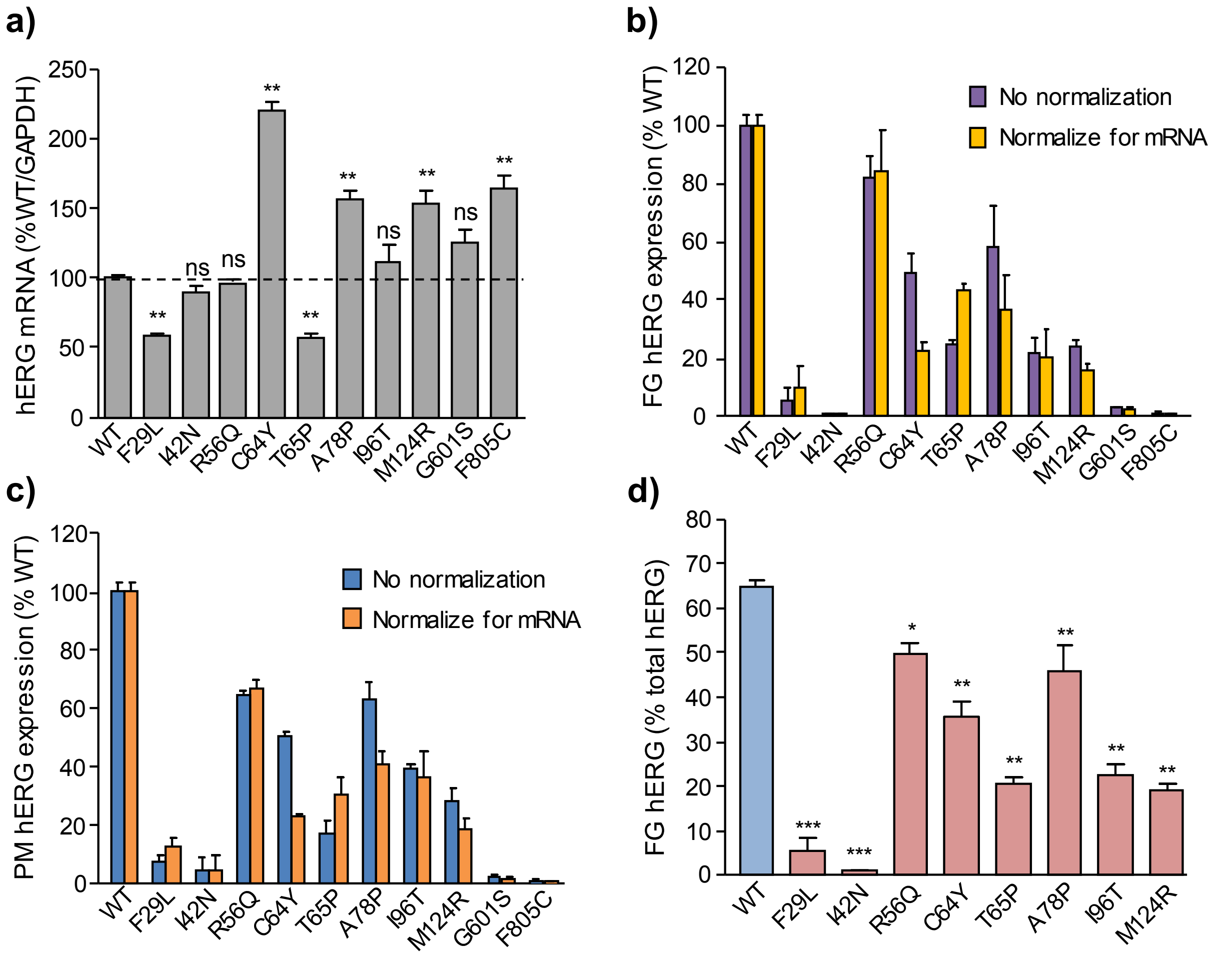

Supplementary Fig. S1: PAS-mutant hERG mRNA and protein expression

(A) hERG transcript levels in stably-expressing HeLa cells. WT and PAS-mutant hERG stably expressed in HeLa cells via lentiviral transduction. mRNA transcript levels determined by qPCR and expressed as % of WT. **(B)** Mature (complex-glycosylated) PAS-mutant hERG protein expression determined by immunoblot analysis and expressed as % of WT-hERG prior to and following normalization for mRNA content. Representative immunoblots and protein expression following normalization for mRNA shown in Fig. 1. **(C)** hERG PM-expression determined by PM-ELISA and expressed as % of WT-hERG prior to and following normalization for mRNA content. PM-expression following normalization for mRNA shown in Fig 1. **(D)** Mature protein hERG expression determined by immunoblot analysis and expressed as fraction of total (mature + immature) hERG protein. * $P < 0.05$, ** $P < 0.01$, *** $P < 0.001$, n.s. indicates no significant difference (See Methods for explanation of statistical analysis).

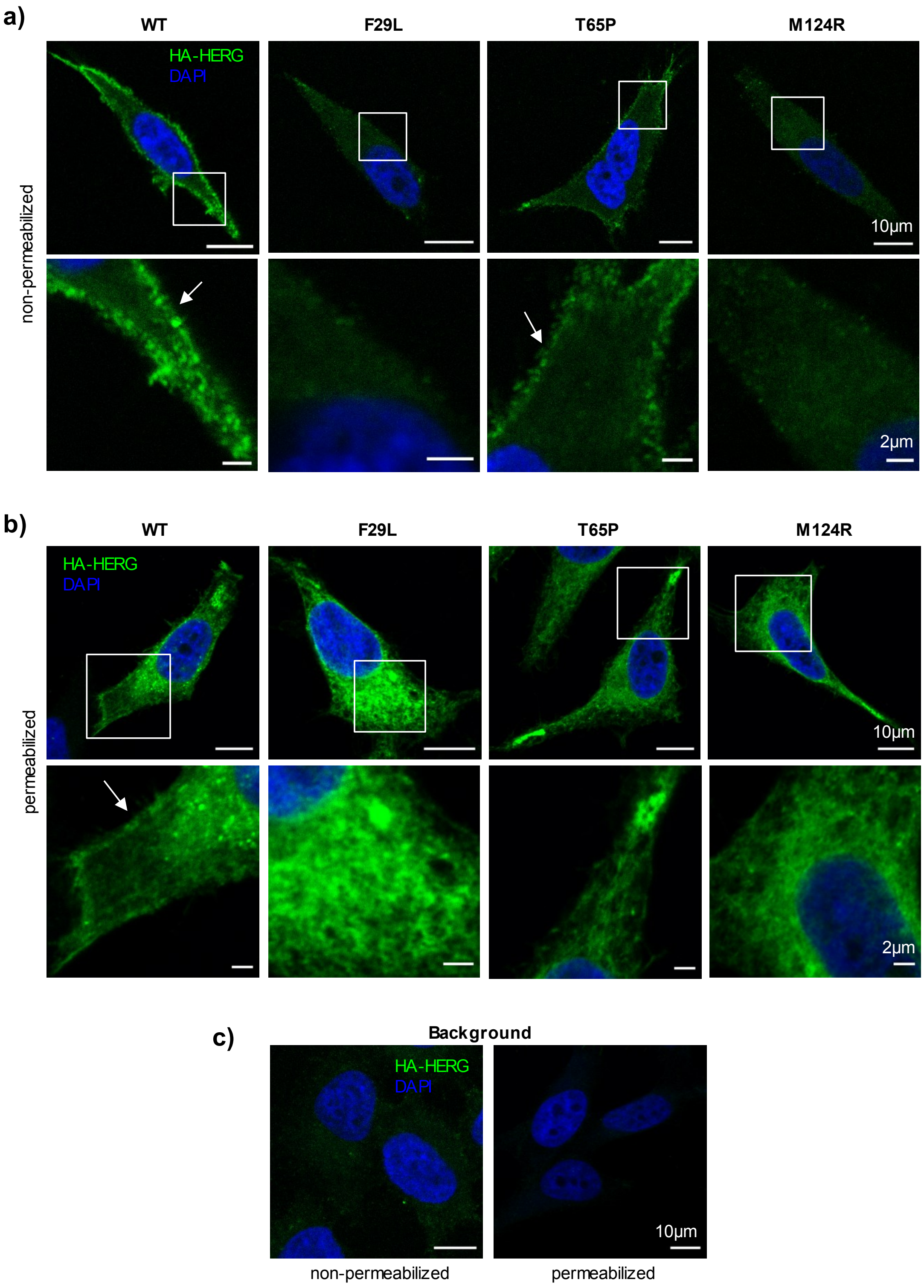

Supplementary Fig. S2: Additional microscopy images of hERG expression and distribution
(figure legend on next page)

Supplementary Fig. S2: Additional microscopy images of hERG expression and distribution (A) PM-hERG pool visualized by confocal immunofluorescence microscopy. PM-hERG labelled via an engineered extracellular HA-epitope tag prior to fixation/permeabilization. Whole-cell (scale bar: 10μM, top) and high-magnification (scale bar: 2μM, bottom) images shown. Magnified area indicated with a white box. WT-hERG shows strong PM distribution (white arrow). Whole-cell images for WT, F29L and T65P hERG shown previously in Fig. 1. **(B)** hERG subcellular distribution visualized by confocal immunofluorescence microscopy following fixation and permeabilization. Whole-cell (scale bar: 10μM, top) and high-magnification (scale bar: 2μM, bottom) images shown. Magnified area indicated with a white box. WT-hERG shows strong PM distribution (white arrow) while select PAS mutants (F29L, T65P and M124R) are mostly confined to intracellular compartments. Whole-cell images for WT, F29L and T65P hERG shown previously in Fig. 1. **(C)** Background fluorescence evaluated in non-expressing HeLa cells. Scale bar: 10μM.

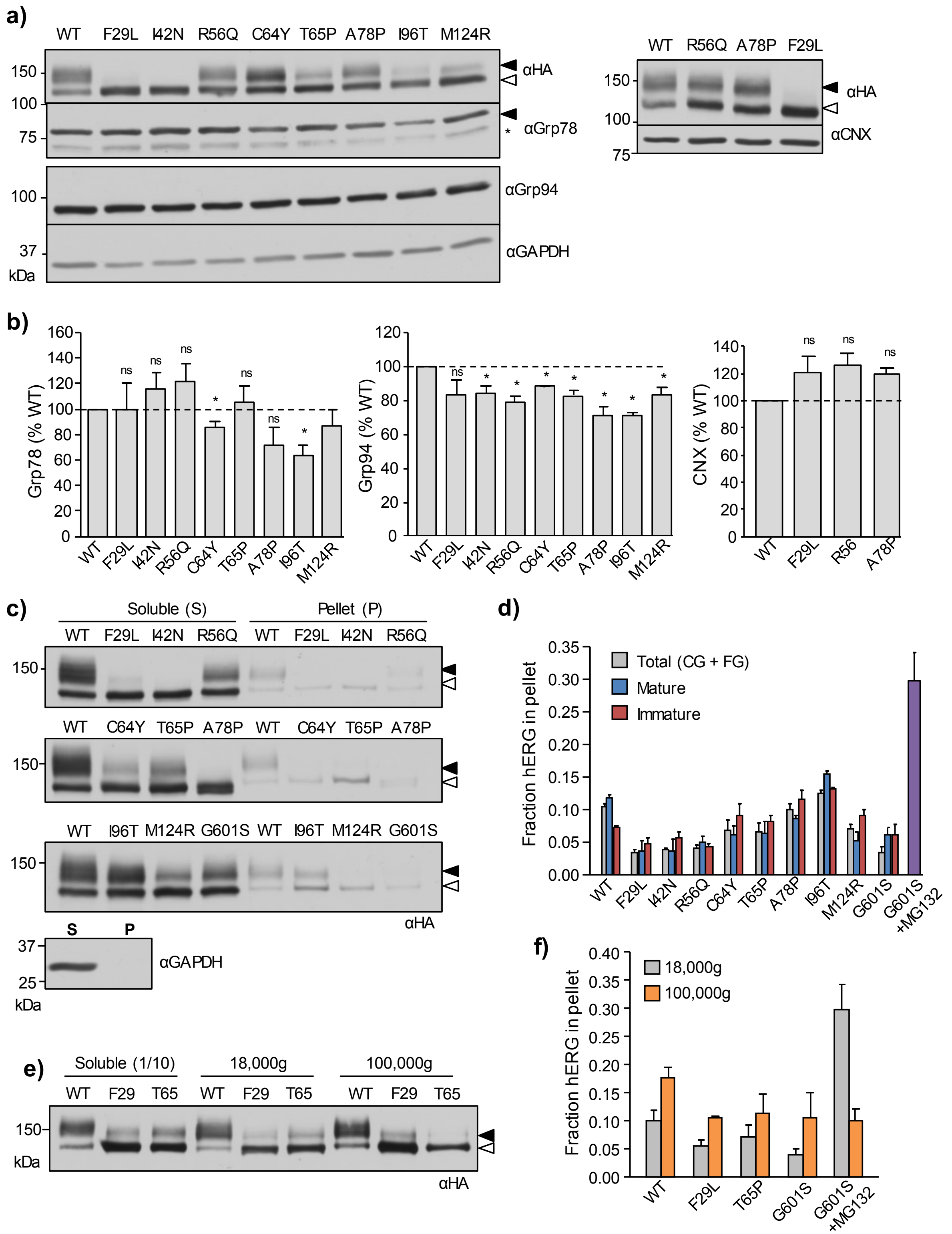

Supplementary Fig. S3: Overexpression of PAS-mutant hERG does not result in aggregation or ER-stress (figure legend on next page)

Supplementary Fig. S3: Overexpression of PAS-mutant hERG does not result in aggregation or ER-stress

(A) Expression of select ER-stress markers (Grp78, Grp94 and Calnexin) evaluated by immunoblot analysis. Asterisk (*) indicated nonspecific band. CNX: Calnexin. GAPDH: loading control **(B)** Densitometric quantification of ER-stress marker expression. Mature (complex-glycosylated) and immature (core-glycosylated) hERG indicated by solid and empty arrow, respectively. Asterisk (*) indicated non-specific band. **(C)** PAS-domain mutations do not increase hERG aggregation propensity. HeLa cells stably expressing HA-tagged hERG solubilized in detergent (1% Triton X-100). Detergent-soluble (S) and insoluble (pellet, P) protein fractions isolated by centrifugation at 18,000g and evaluated by immunoblotting. Equal fractions of soluble (S) and pellet (P) were loaded. GAPDH: soluble protein control. **(D)** Densitometric quantification of mature, immature, and total (mature + immature) hERG isolated from detergent-insoluble pellet. Detergent-insoluble hERG expressed as a fraction of total (S+P) hERG. Positive control: mutant (G601S) hERG-expressing HeLa cells treated overnight with MG132 (1μM, purple). **(E-F)** Similar results were obtained when whole-cell lysates were subject to high-speed centrifugation (100,000g vs. 18,000g). To aid densitometric quantification, only 1/10th of soluble fraction was loaded relative to 18,000g and 100,000g pellets. * $P < 0.05$, ** $P < 0.01$, *** $P < 0.001$, n.s. indicates no significant difference (See Methods for explanation of statistical analysis). Representative immunoblots shown (uncropped images in Supplemental Fig. S13). Solid line indicates different parts of the same gel. White space indicates separate gels.

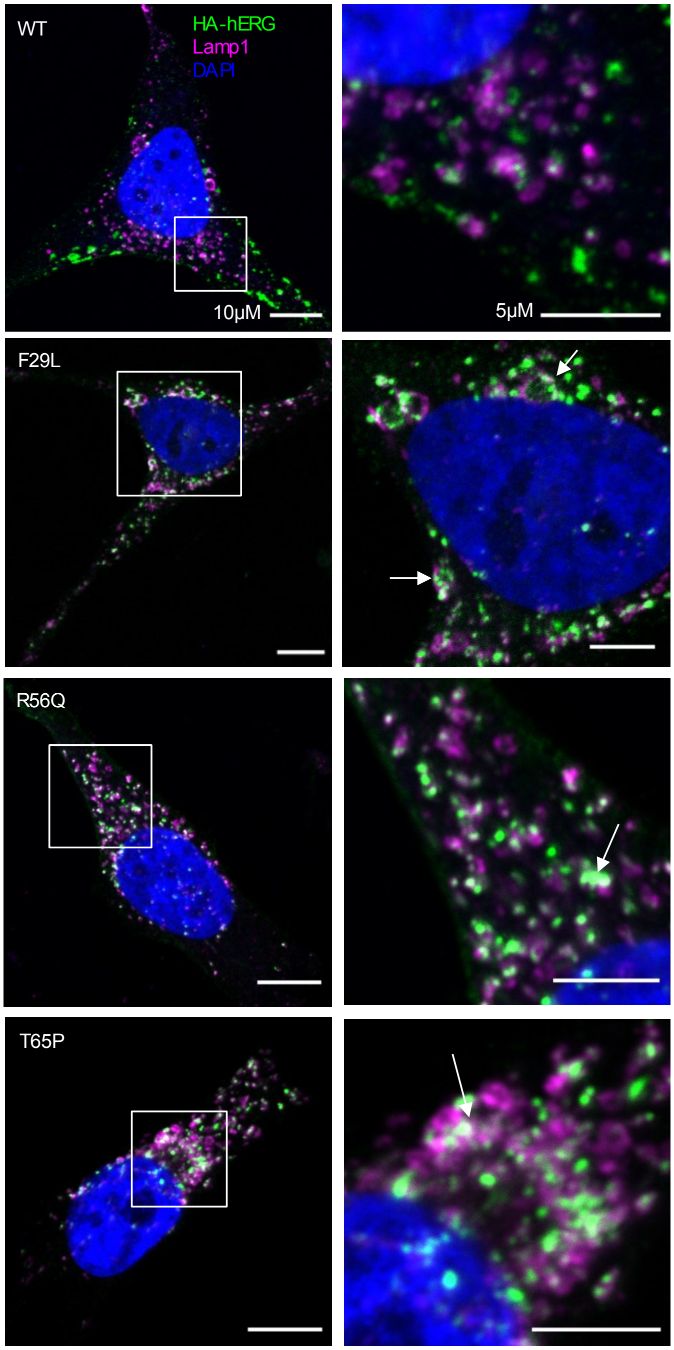

Supplementary Fig. S4: Additional microscopy images of hERG lysosomal delivery
Post-endocytic lysosomal delivery of additional PAS-mutant hERG (F29L, R56Q and T65P) evaluated by confocal immunofluorescence microscopy. Endocytic hERG pool labelled by Ab capture (15min at 37°C) and remaining cell-surface hERG blocked with unconjugated secondary F'ab (1h on ice). Cells then chased at 37°C for 3h prior to fixation. Lysosomal compartments labelled with LAMP1 pAb. hERG (green) and LAMP1 (magenta) staining visualized by laser confocal microscopy. Whole-cell (scale bar: 10μM, left) and high-magnification (scale bar: 5μM, right) images shown. Magnified area indicated by white box. Representative control (WT-hERG) images previously shown in Fig. 4.

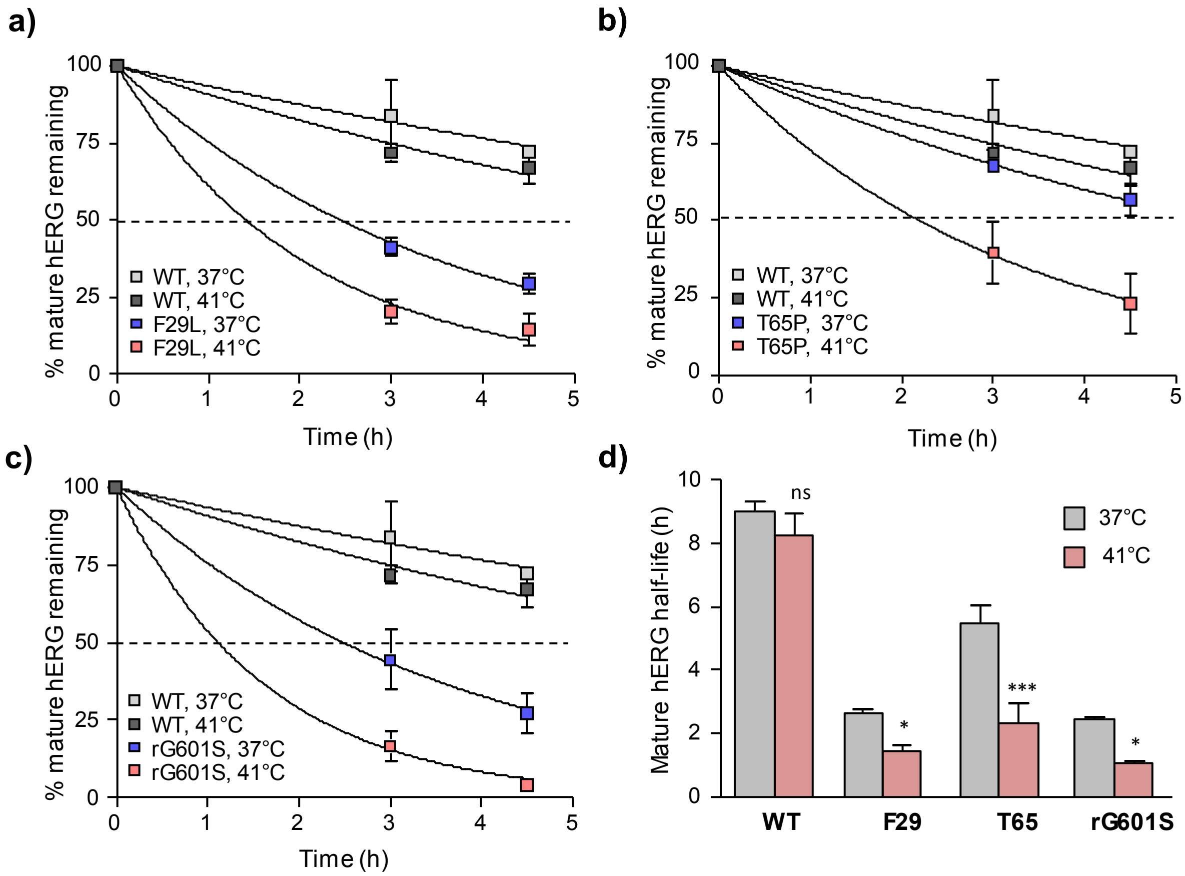

Supplementary Fig. S5: Thermosensitivity of PAS-mutant hERG mature protein turnover (A-D) Metabolic stability of WT, PAS-mutant (F29L and T65P) or temperature-rescued G601S (48h at 26°C, rG601S) hERG evaluated at 37°C or 41°C by immunoblotting following translational inhibition with cycloheximide (CHX, 150μg/ml). Mature hERG turnover kinetics fit as a single-exponential decay function to calculate half-lives shown in (D). Representative immunoblots and densitometric quantification of WT and F29L hERG turnover shown previously in Fig. 5. * P < 0.05, ** P < 0.01, *** P < 0.001, n.s. indicates no significant difference (See Methods for explanation of statistical analysis).

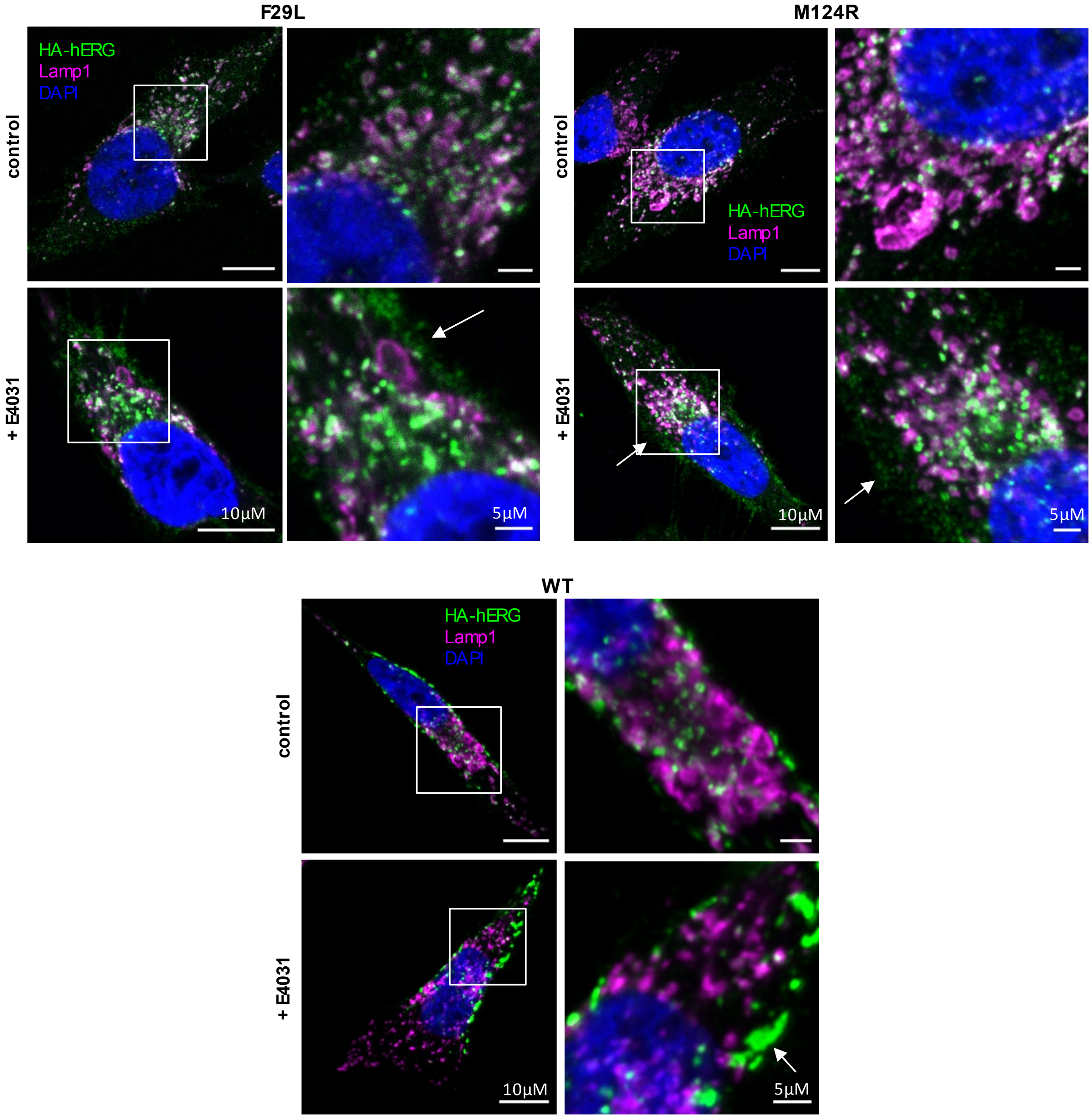

Supplemental Fig. S6: Additional microscopy images of hERG lysosomal delivery
Post-endocytic lysosomal delivery of additional PAS-mutant (F29L and M12R) and WT-hERG evaluated by confocal immunofluorescence microscopy following overnight pre-treatment with E4031 (10µM). Endocytic hERG pool labelled by Ab capture (15min at 37°C) and remaining cell-surface hERG blocked with unconjugated secondary F'ab (1h on ice). Cells then chased at 37°C for 3h prior to fixation. Lysosomal compartments labelled with LAMP1 pAb. hERG (green) and LAMP1 (magenta) staining visualized by laser confocal microscopy. Whole-cell (scale bar: 10µM, left) and high-magnification (scale bar: 5µM, right) images shown. Magnified area indicated by white box.

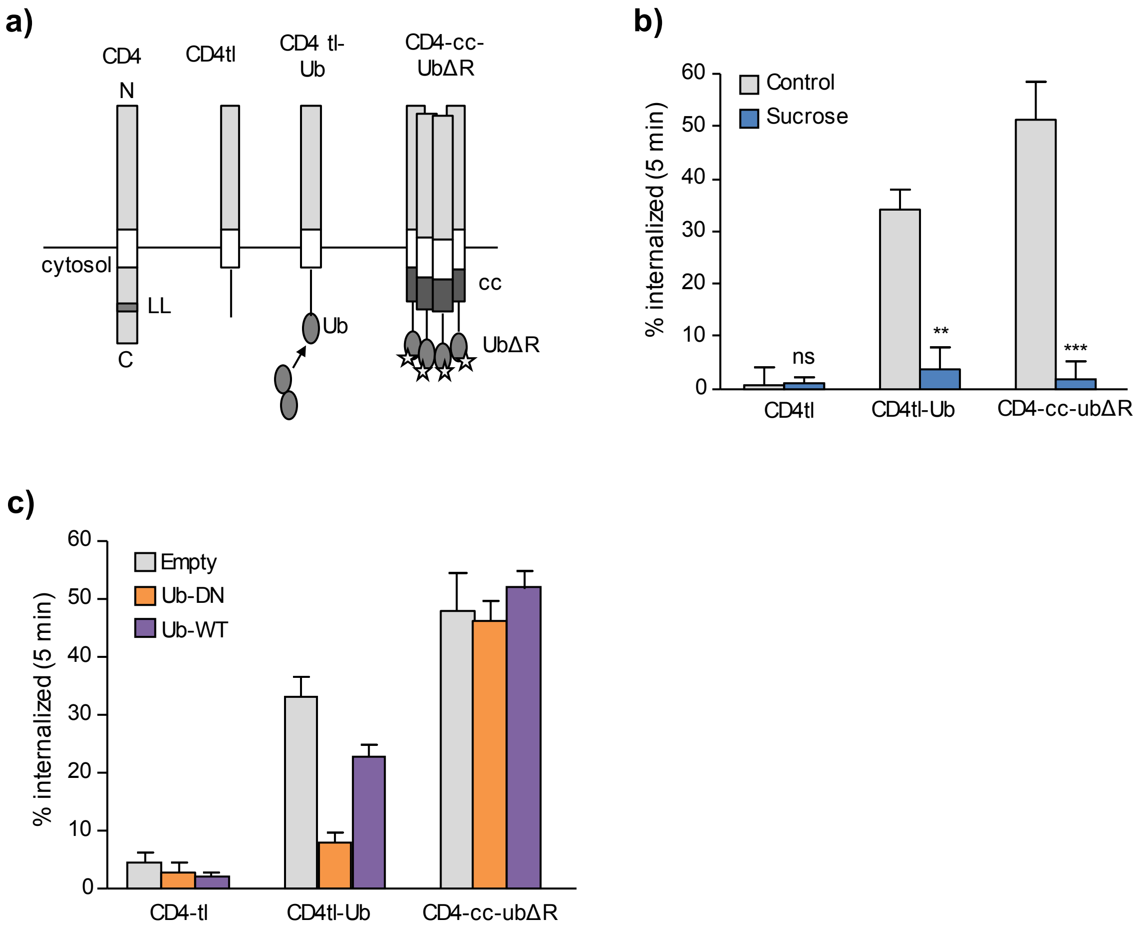

Supplementary Fig. S7: Inhibition of ubiquitination and clathrin-dependent internalization **(A)** Domain structure of CD4-chimeric constructs. Native CD4 is a single-span transmembrane protein with an endogenous dileucine signaling motif (LL). CD4tl: endogenous cytosolic tail replaced by flexible linker. CD4tl-ub: CD4-tl construct with C-terminal fused ubiquitin (ub) which supports polyubiquitination. CD4-cc-ubΔR: coiled-coil (cc) tetramerization motif and C-terminal fused lysine-free ubiquitin (ubΔR). Lys-less ubiquitin does not support polyubiquitination, but tetramerized construct mimics multi-mono and poly-ubiquitination[1,2]. (**B)** Inhibition of clathrin-dependent internalization prevents internalization of CD4-ubiquitin chimeras. Clathrin-dependent endocytosis inhibited by hypertonic shock in media supplemented with 300mM sucrose (15min at 37°C followed by 45min at 4°C). Amount of CD4 internalized during 5-minutes measured using cell-surface ELISA. **(C)** Overexpression of dominant-negative ubiquitin suppresses polyubiquitination of CD4-tl-ubi. CD4 constructs coexpressed with excess empty vector, wild-type ubiquitin (ub-WT) or lys-free ubiquitin unable to support formation of linked chains (ub-DN). Overexpression of ub-DN but not the WT ubiquitin prevented the internalization of CD4-tl-ub, presumably by inhibiting formation of linked chains. Internalization of CD4-cc-ubΔR, which mimics polyubiquitinated cargo yet is unsusceptible to linked chain polyubiquitination remains unaffected. * $P < 0.05$, ** $P < 0.01$, *** $P < 0.001$, n.s. indicates no significant difference (See Methods for explanation of statistical analysis).

a)

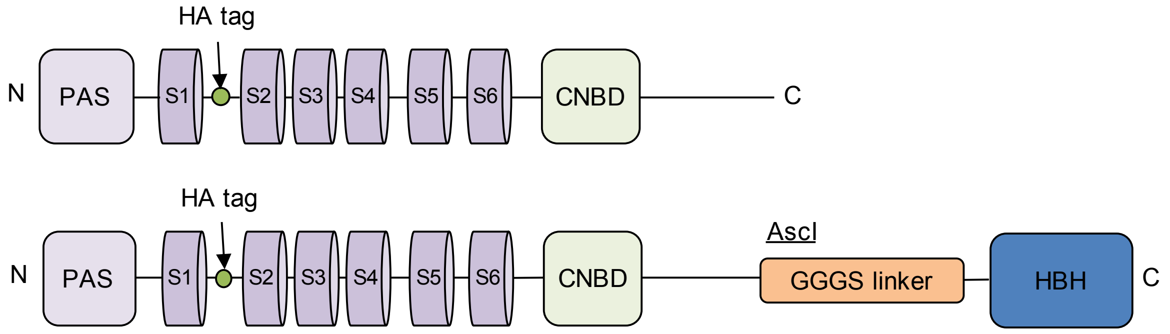

b)

…ccg ggc agt ggc gcg cca gga gga ggt ggg tct gga ggt gga gga tcc ggt ggg ggt ggg tct cat cat cac cac cat cat gct gga aag gcc ggt gaa ggt gaa atc cct gcc cct ctt gct ggt acc gtt tct aag ata ctg gta aaa gaa ggt gac act gtt aaa gct gt caa aca gtt ctg gtg ctg gag gct atg aaa atg gag aca gaa att aac gct cct act gac gga aaa gtt gaa aag gtg tta gtt aag gaa aga gat gct gtt caa ggt ggt caa ggt cta atc aag atc ggc gtt cat cat cac cac cat cat taa tga

…PGSGAPGGGGSGGGGSGGGGSHHHHHHAGKAGEGEIPAPLAGTVSKILVKEGDTVKAGQTVLVLEAMKM ETEINAPTDGKVEKVLVKERDAVQGGQGLIKIGVHHHHHH**

Supplementary Fig. S8: C-terminal HBH-tagged hERG constructs
(A) Domain structure of HA-tagged hERG (top) and C-terminal HBH-tagged construct. Indicated are the engineered HA epitope tag[5], GGGS linker (including engineered AscI cut-site) and his-biotin-his (HBH) tag. **(B)** cDNA (top) and protein sequence (bottom) of additional linker and HBH tag regions. Black: hERG C-terminus region. Orange: GGGS linker including AscI cut site (underlined). Blue: HBH tag coding sequence.

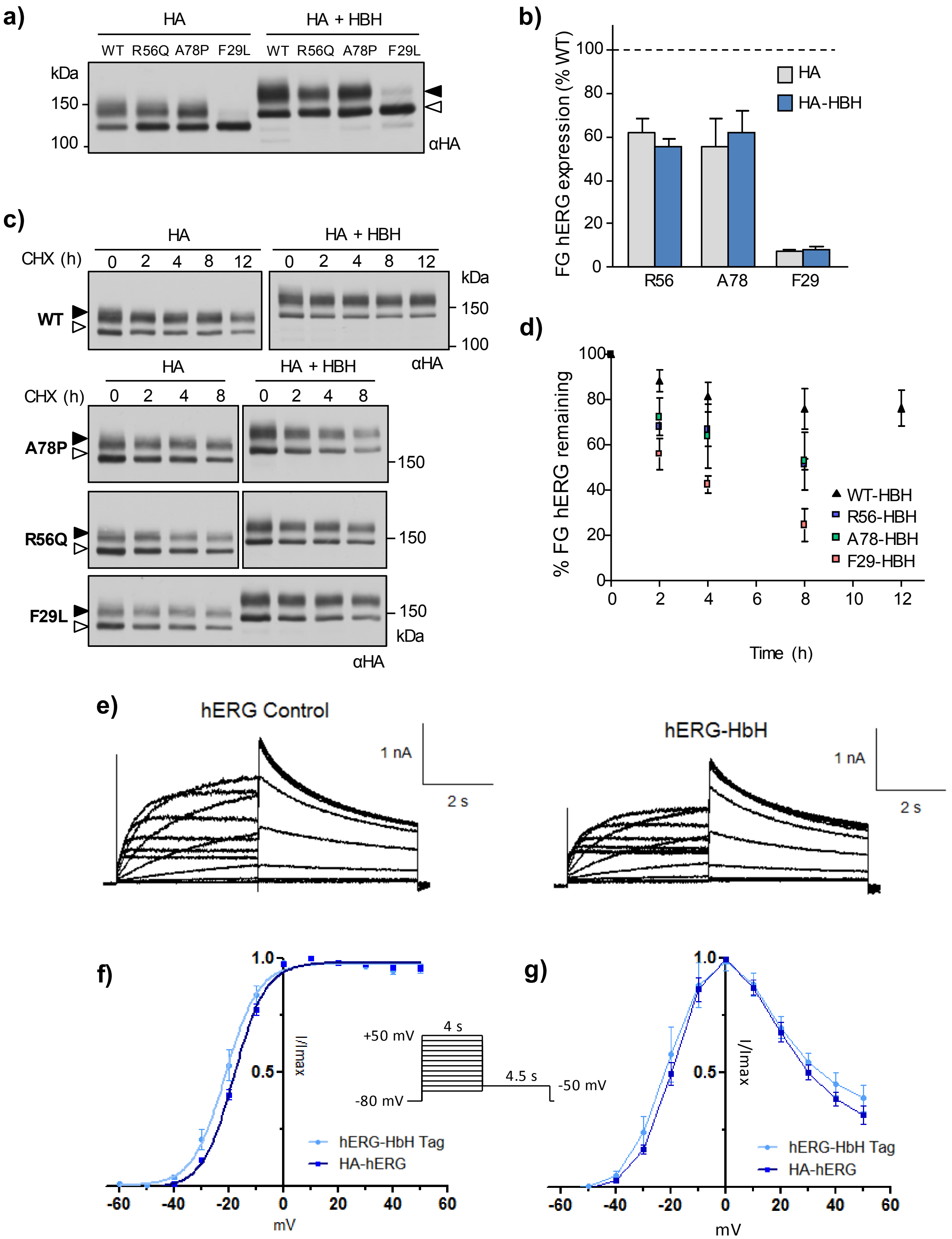

Supplemental Fig. S9: HBH tag does not alter hERG cellular processing or function
(figure legend on next page)

Supplemental Fig. S9: HBH tag does not alter hERG cellular processing or function

(A) Protein expression of HA-tagged and C-terminus HBH-tagged WT and PAS-mutant hERG assayed by immunoblotting. Note apparent molecular-mass shift due to addition of HBH tag. (**B)** Quantification of mature hERG expression. Mature (complex-glycosylated) and immature (core-glycosylated) hERG indicated by solid and empty arrow, respectively. **(C)** Metabolic turnover of mature WT and PAS-mutant HA- and HBH-tagged hERG assayed by immunoblotting following translational inhibition with cycloheximide (CHX, 150µg/ml). **(D)** Quantification of mature hERG turnover. **(E)** Representative traces of WT-hERG (left) and WT-hERG-HBH (right) evoked from a holding potential of -80 mV, stepped in +10 mV depolarizing pulses from -60 mV to +50 mV for 4 seconds (P1 pulse), and subsequently stepped to -50 mV for 4.5 seconds (P2 pulse). **(F)** Steady state activation curve obtained from a plot of normalized P2 peak tail currents (at -50 mV) against P1 voltages for WT-hERG (squares) and WT-hERG-HBH (circles). Normalized plots (mean ± SEM) were fit with a Boltzmann function. **(G)** Steady state current voltage relationship obtained from a plot of normalized P1 peak currents against P1 voltages for WT-hERG (squares) and WT-hERG-HBH (circles). Normalized plots are presented as mean ± SEM. No statistically-significant difference was found in data shown in (F) and (G), as determined by a paired two-tailed T-test. In addition, the curves describing the kinetics of hERG current activation and inactivation were found to be indistinguishable (data not shown). Representative immunoblots shown (uncropped images in Supplemental Fig. S13). Solid line indicates different parts of the same gel. White space indicates separate gels.

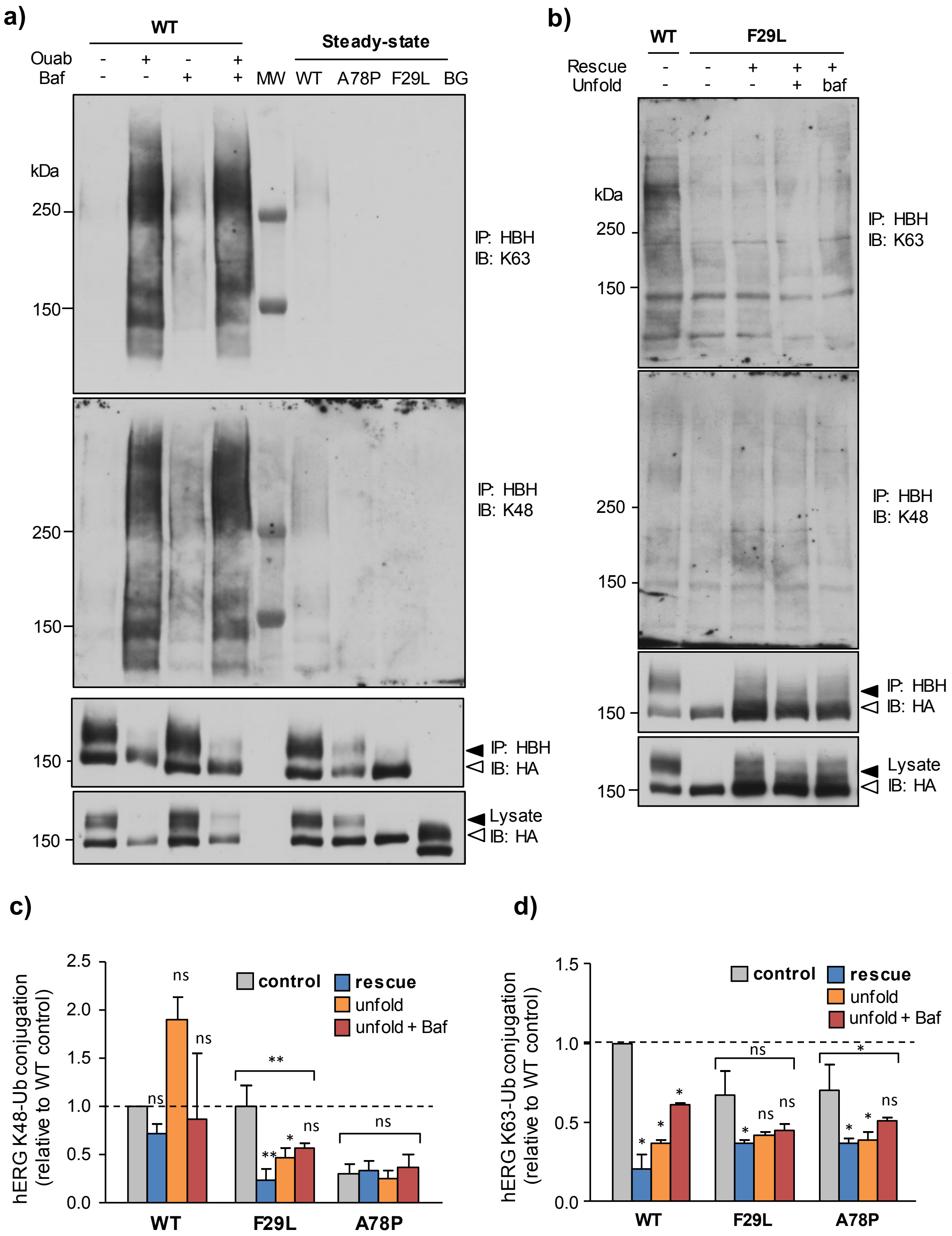

Supplementary Fig. S10: PAS mutant hERG do not undergo significant ubiquitination
(Figure legend on next page)

Supplementary Fig. S10: PAS mutant hERG do not undergo significant ubiquitination
(A) PAS mutant hERG do not undergo K48 or K63 linked-chain polyubiquitination under steady-state conditions. hERG-HBH were affinity-isolated on monomeric avidin beads. Ubiquitination detected by immunoblotting with K48 and K63 linked-chain specific monoclonal Ab (See Methods and Materials). WT-hERG destabilized by acute intracellular K^+ depletion with ouabain (ouab, 3h at 300nM) displayed marked ubiquitination (left). In contrast, PAS-mutant channels did not display a similar increase in ubiquitination (right). Non-specific binding assessed in HeLa cells expressing non-HBH-tagged WT-hERG (rightmost lane). Baf: Bafilomycin A1 (200nM). MW: molecular-weight marker non-specifically recognized by αK48-Ub and αK63-Ub Abs. Mature (complex-glycosylated) and immature (core-glycosylated) hERG indicated by solid and empty arrow, respectively. **(B-D)** Attenuated PAS-mutant hERG ubiquitination is not dependent on mature protein expression. Cells expressing hERG-HBH were subject to low-temperature rescue (26°C for 24h) and subsequent unfolding (37°C for 3h) in the presence/absence of Bafilomycin A1 (Baf, 200nM). hERG was affinity-isolated and K48/K63 ubiquitination detected by immunoblotting (B) or ELISA (C-D) using linked-chain specific monoclonal Ab (See Methods and Materials). Ubiquitination expressed as fold increase relative to untreated WT-control. MW: molecular-weight marker non-specifically recognized by αK48-Ub and αK63-Ub Abs.* $P < 0.05$, ** $P < 0.01$, *** $P < 0.001$, n.s. indicates no significant difference (See Methods for explanation of statistical analysis). Representative immunoblots shown. Immunoblots for ubiquitin are not cropped. Uncropped αHA blots in Supplemental Fig. S12. Solid line: different parts of the same gel. White space: separate gels. Anti-HA immunoblots are the same as those shown in Fig. 7.

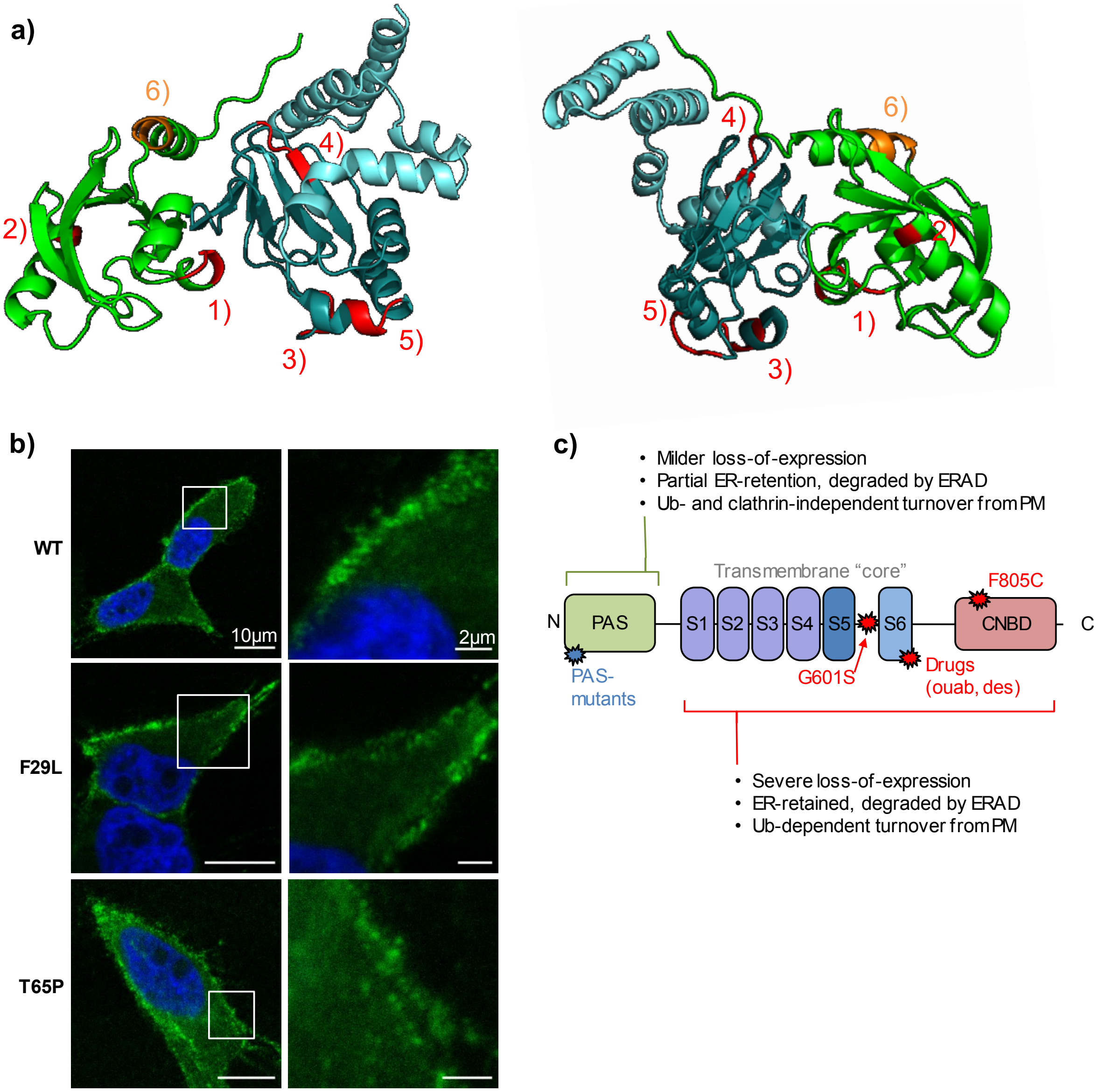

Supplementary Fig. S11: Potential internalization/sorting signals for PAS-mutant hERG
(A) Signalling sequences in hERG cytosolic domains. The hERG PAS/CNBD domains contain 5 tyrosine-based sorting motifs (red) and a KFERQ-related sequence (orange). Signalling sequences summarized in Supplementary Table T3. **(B)** PAS mutant hERG do not appear to aggregate at the cell-surface. Cell-surface hERG labelled using HA antibody on-ice prior to fixation and permeabilization. PAS-mutant (F29L and T65P) hERG imaged with higher detector gain in order to visualize cell-surface distribution. Cell-surface staining of PAS-mutant hERG was notably weaker than WT (Fig. 1) but the distribution pattern was not appreciably different. Whole-cell (scale bar: 10µM) and high-magnification (scale bar: 2µM) images shown. Magnified area indicated by white box. **(C)** Overview of hERG mutation-specific proteostatic processing described in this study. The location of PAS-domain mutations (blue), severe ER-retained mutations (G601S and F805C, red) and drug-induced disruption of WT-hERG by ouabain (ouab) and desipramine (des) indicated on the hERG1a domain structure. The observed PM-expression and stability and ER-processing phenotype are noted.

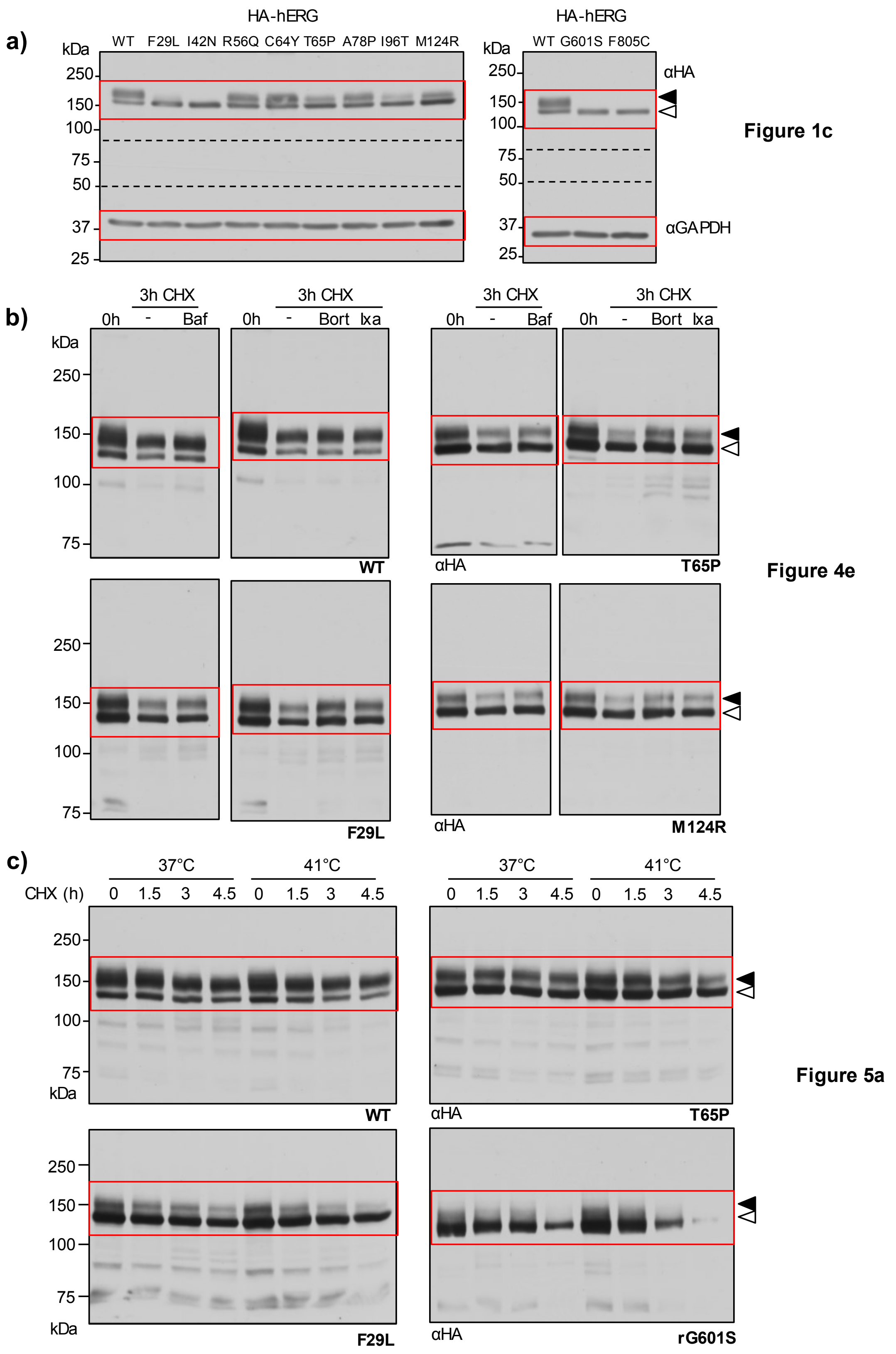

Supplementary Fig. S12: Full-length (uncropped) images (main figure set)

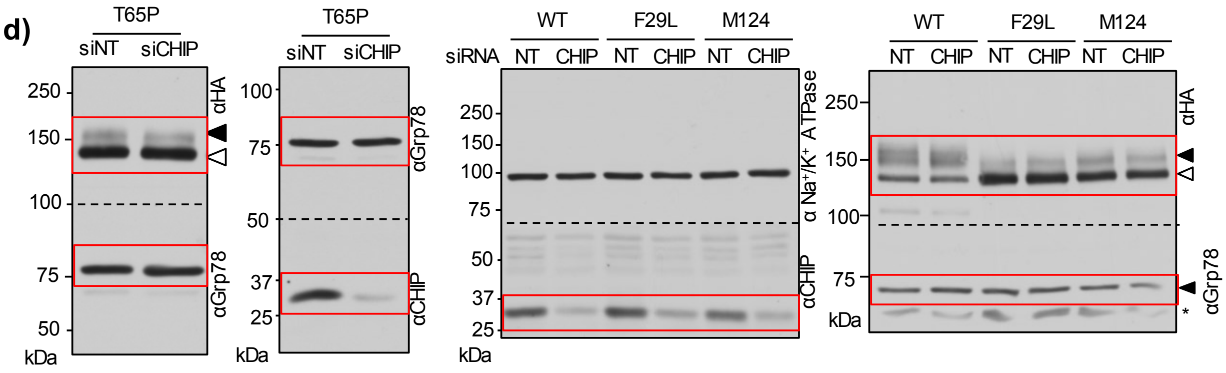

Figure 6b

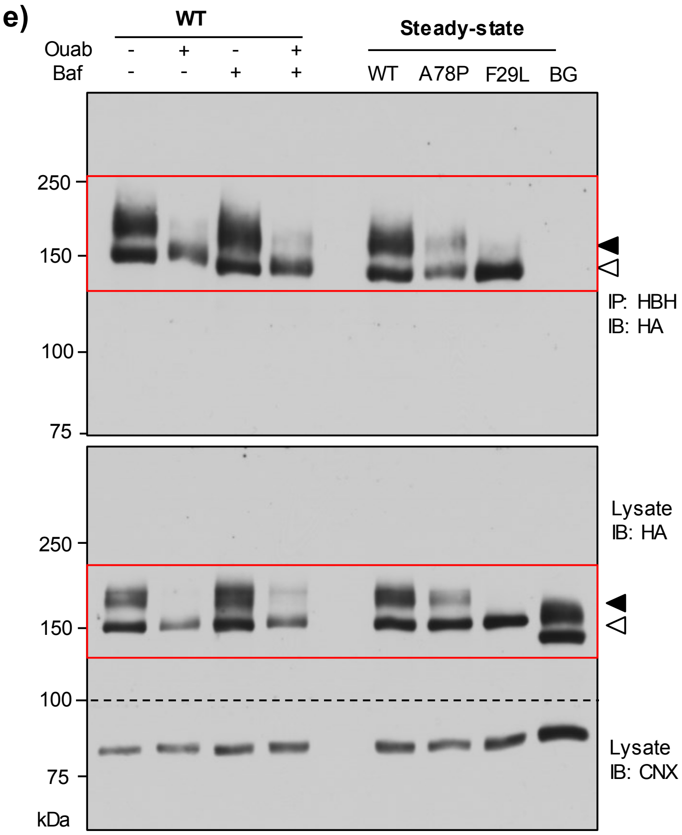

Figure 7a and S10

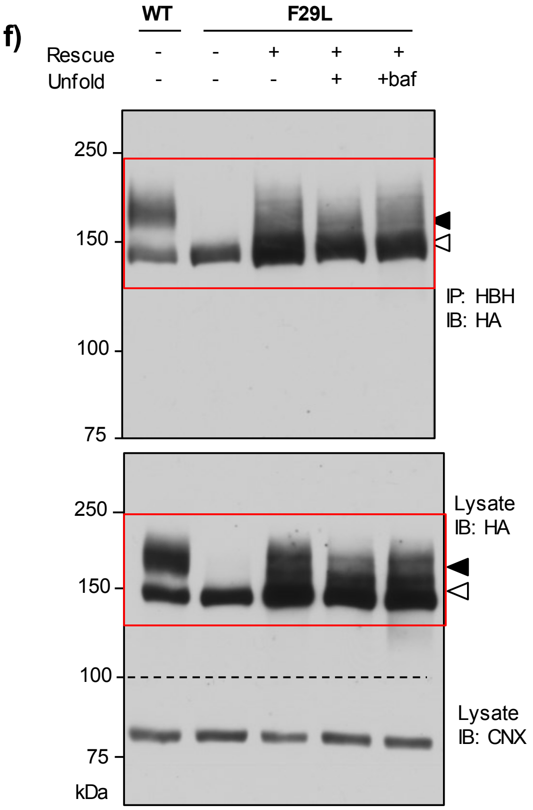

Figure 7c and S10

Supplementary Fig. S12 (continued)

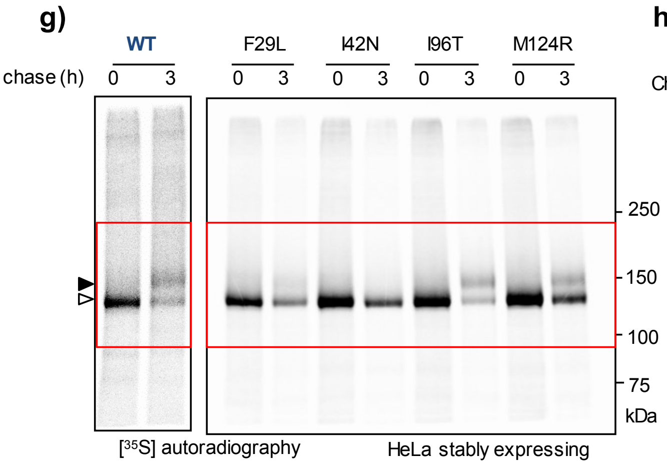

Figure 2a

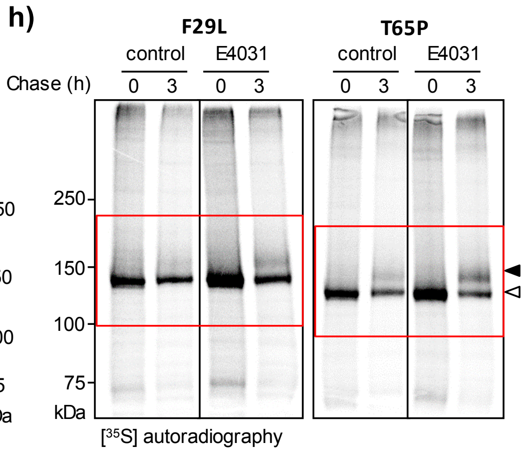

Figure 2c

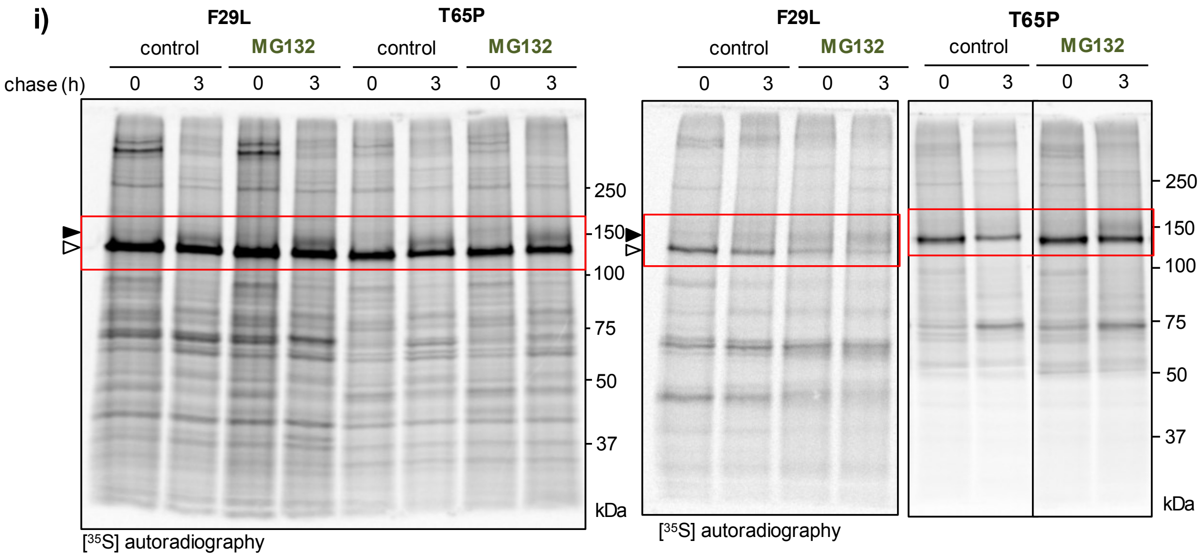

Figure 2e

Supplementary Fig. S12: Full-length (uncropped) images (main figure set)
Uncropped immunoblots (A-F) and autoradiograms (G-I). Where applicable, immature core-glycosylated (~135kDa) and mature complex-glycosylated (~155kDa) hERG indicated with empty and solid arrows, respectively. Asterisk (*) indicated nonspecific band. Solid line: different parts of the same gel. White space: separate gels. Dotted line: location where membrane was cut for blotting multiple substrates. Portion of image used in the figure indicated by red box. CNX: Calnexin.

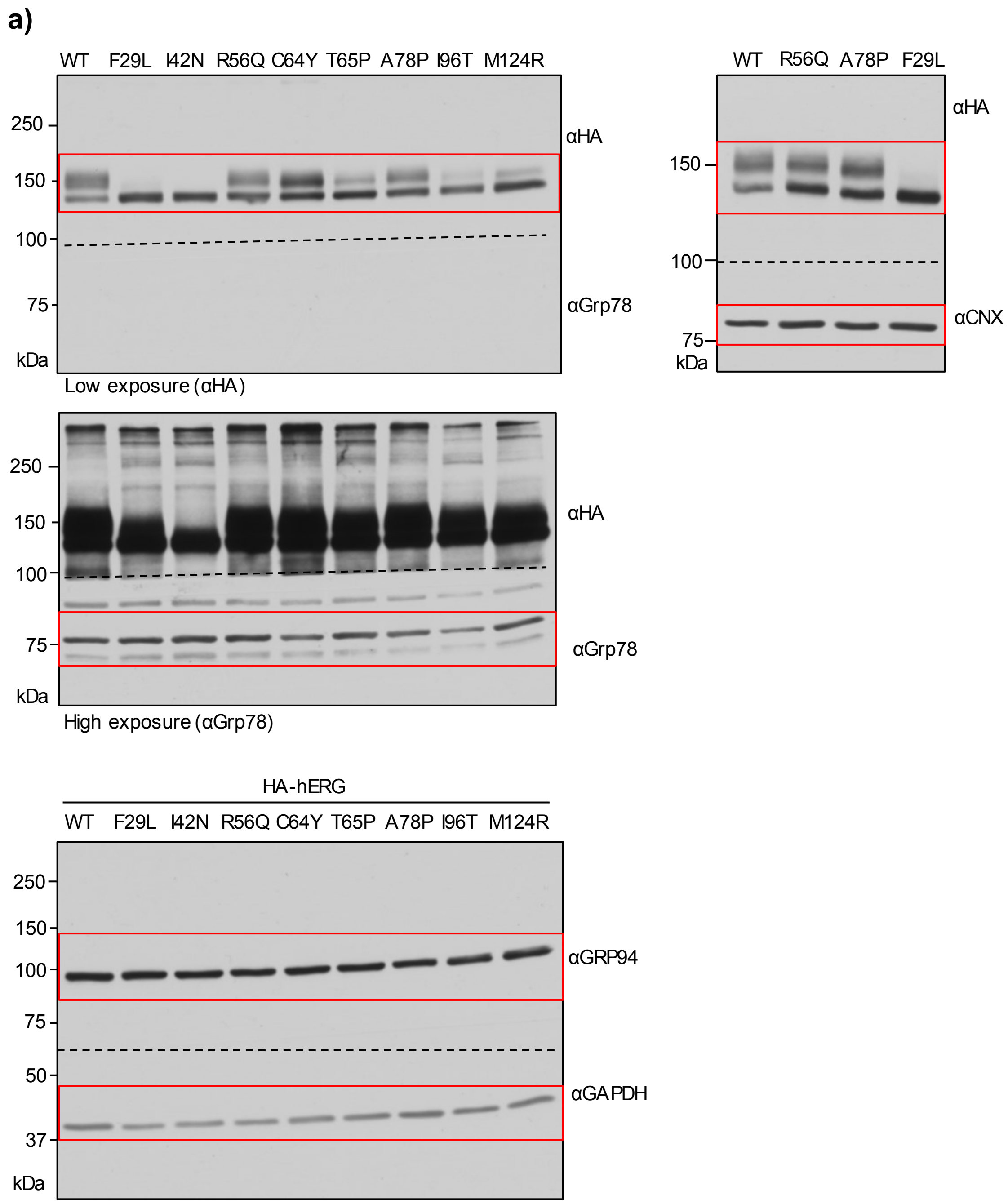

Supplemental Fig. S3a

Supplementary Fig. S13: Full-length (uncropped) images (supplemental figure set)

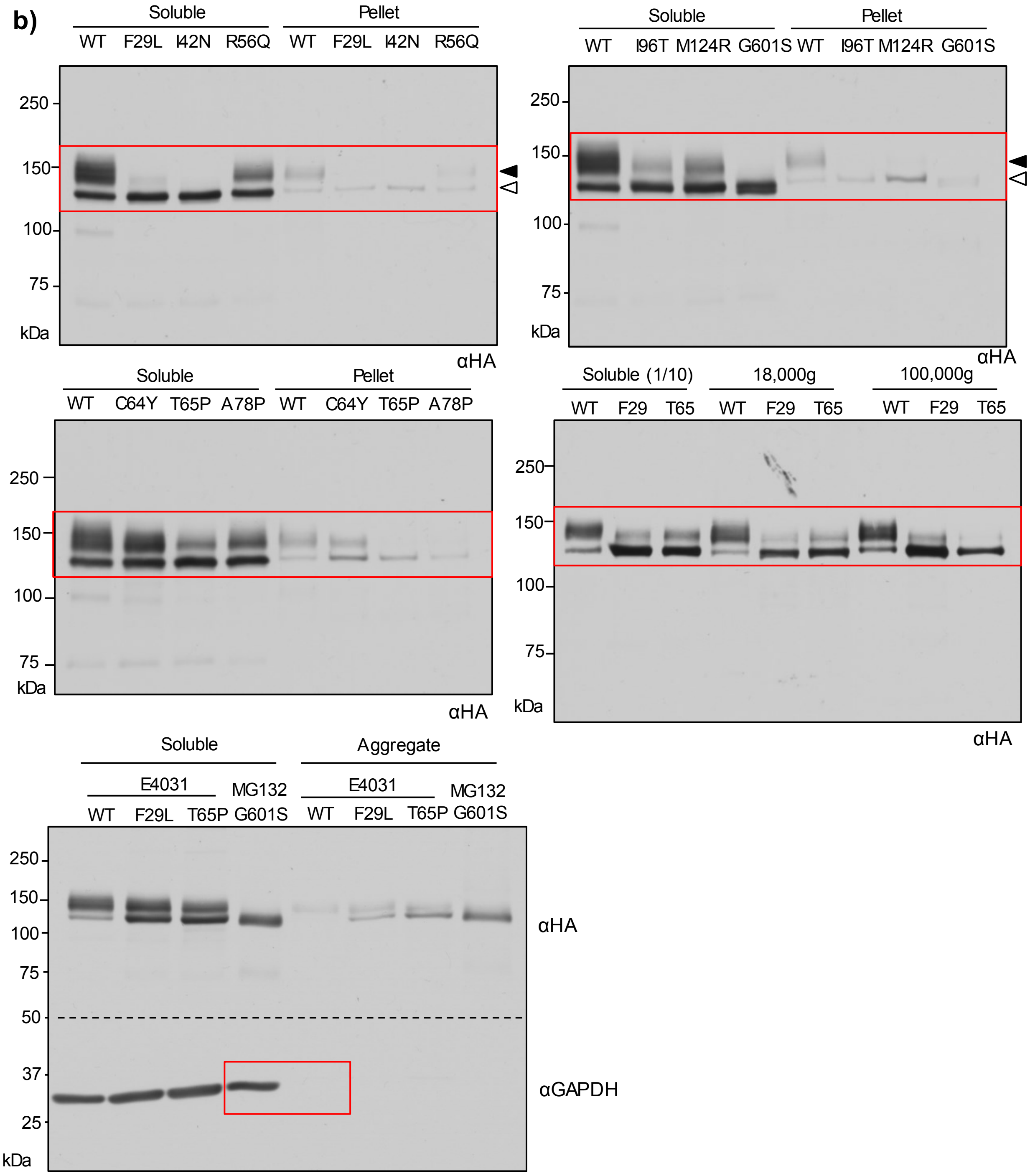

Supplemental S3c-e

Supplementary Fig. S13 (continued)

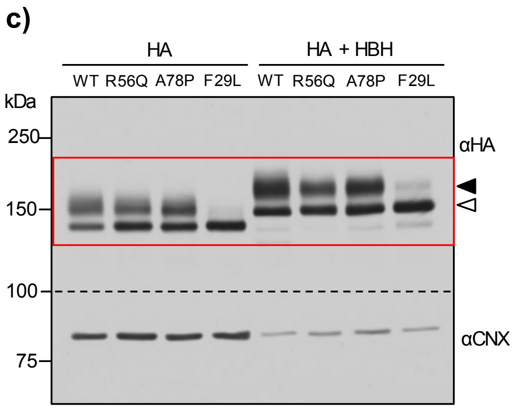

Supplemental Fig. S9a

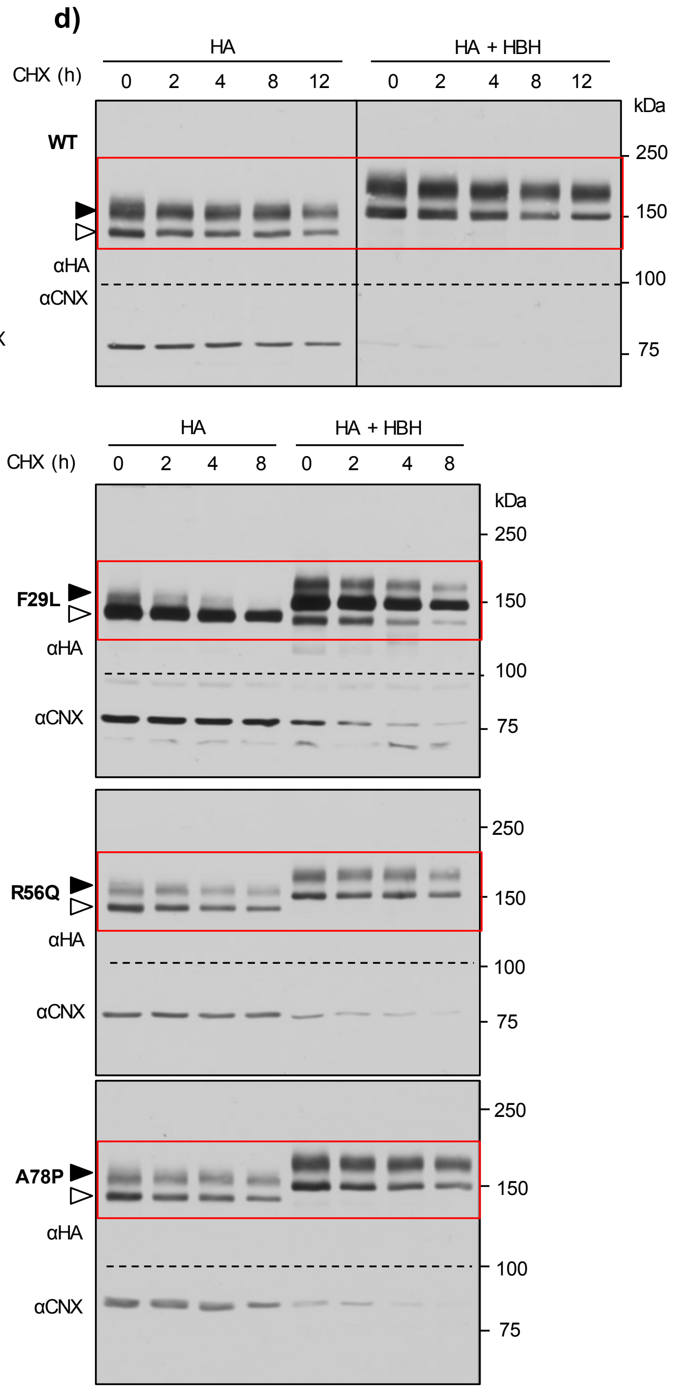

Supplemental Fig. S9c

Supplementary Fig. S13: Full-length (uncropped) images (supplemental figure set)
Uncropped immunoblot images. Where applicable, immature core-glycosylated (~135kDa) and mature complex-glycosylated (~155kDa) hERG indicated with empty and solid arrows, respectively. Asterisk (*) indicated nonspecific band. Solid line: different parts of the same gel. White space: separate gels. Dotted line: location where membrane was cut for blotting multiple substrates. Portion of image used in the figure indicated by red box. CNX: Calnexin.
